# Cell Sources for Retinal Regeneration: Implication for Data Translation in Biomedicine of the Eye

**DOI:** 10.3390/cells11233755

**Published:** 2022-11-24

**Authors:** Eleonora N. Grigoryan

**Affiliations:** Koltzov Institute of Developmental Biology, Russian Academy of Sciences, 119334 Moscow, Russia; leonore@mail.ru

**Keywords:** retinal degenerative diseases, retinal regeneration, intrinsic cell sources, regulatory network, ophthalmotherapy

## Abstract

The main degenerative diseases of the retina include macular degeneration, proliferative vitreoretinopathy, retinitis pigmentosa, and glaucoma. Novel approaches for treating retinal diseases are based on cell replacement therapy using a variety of exogenous stem cells. An alternative and complementary approach is the potential use of retinal regeneration cell sources (RRCSs) containing retinal pigment epithelium, ciliary body, Müller glia, and retinal ciliary region. RRCSs in lower vertebrates in vivo and in mammals mostly in vitro are able to proliferate and exhibit gene expression and epigenetic characteristics typical for neural/retinal cell progenitors. Here, we review research on the factors controlling the RRCSs’ properties, such as the cell microenvironment, growth factors, cytokines, hormones, etc., that determine the regenerative responses and alterations underlying the RRCS-associated pathologies. We also discuss how the current data on molecular features and regulatory mechanisms of RRCSs could be translated in retinal biomedicine with a special focus on (1) attempts to obtain retinal neurons de novo both in vivo and in vitro to replace damaged retinal cells; and (2) investigations of the key molecular networks stimulating regenerative responses and preventing RRCS-related pathologies.

## 1. Introduction

Degenerative changes in the animal and human retina related with age or diseases cause visual dysfunction and blindness. The major, well-known retinal disorders are age-related macular degeneration (AMD), retinitis pigmentosa (RP), and glaucoma. This list also includes diabetic retinopathy, proliferative vitreoretinopathy (PVR), Stargardt disease, etc. One opinion is that retinal degeneration, which leads to decreased vision and blindness, is a health problem comparable in prevalence and significance to Alzheimer’s disease and cancer [1,2].

**Figure 1:**
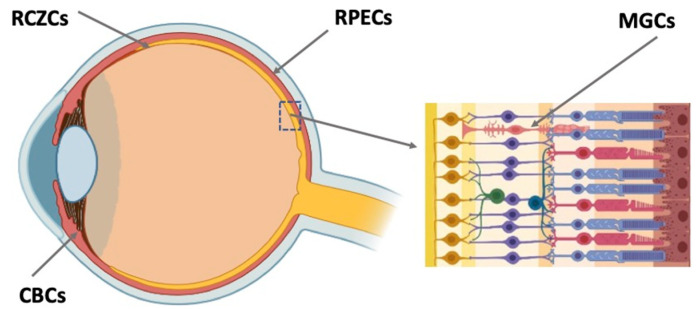
Potential endogenous cell sources for retinal regeneration (summarized data). RCZCs—cells of the retina ciliary zone; RPECs—retinal pigment epithelial cells; CBCs—cells of the ciliary body; MGCs—Müller glial cells.

The long-term search for potential RRCSs and comprehensive investigations have led to the discovery of such sources in the eye tissues and to understanding of their biology. There has been progress in the study of genetic intrinsic features of RRCSs and external regulation mechanisms responsible for retention or acquisition of the low-differentiated state of RRCSs, conversion and neural/retinal differentiation. Despite significant differences in the degree of manifestation of RRCSs’ regenerative potencies in vertebrates in the evolutionary series, all of them, one way or another, belong to the same spectrum (Figure 1). The latter comprises undifferentiated or poorly differentiated precursors localized in the ciliary region of the retina, retinal pigmented epithelium (RPE), ciliary body (CB) of the eye, and also Müller glial cells (MGCs). Note that these cell populations are beyond the set of neural cell phenotypes of the retina, but they are mandatory and integrated in the system of its functions. All of these RRCS types that can potentially be an intrinsic regenerative reserve in the retina are considered in numerous reviews summarizing the results of long-term experimental research conducted by several laboratories worldwide [3,4,5,6,7,8,9,10,11,12]. In the present study, efforts were focused on finding ways for practical application of the accumulated knowledge about RRCSs and approaches to regulating their properties and behavior for the purpose of translation to biomedicine of the eye. In this review, an attempt is made to understand the implications for the development of this research trend.

Currently, embryonic progenitors and embryonic, neural, mesenchymal, and induced pluripotent stem cells are considered to be sources of RPE and neural retina (NR) regeneration, as an alternative to autologous mammalian and human RRCSs. Their use provides a wide range of opportunities, while nevertheless posing numerous risks, as is widely discussed in the modern literature [12,13,14,15,16,17]. Thus, an approach based on transplantation of stem cell-derived RPE cells for the treatment of AMD is currently being developed, with clinical trials conducted [18,19,20,21,22]. Being extensively analyzed and discussed in the literature, this topic is beyond the scope of the present review, which mainly provides information about intrinsic RRCSs and evaluates the feasibility of using this information in biomedicine.

## 2. The Main Structure of the Retina

**Figure 2:**
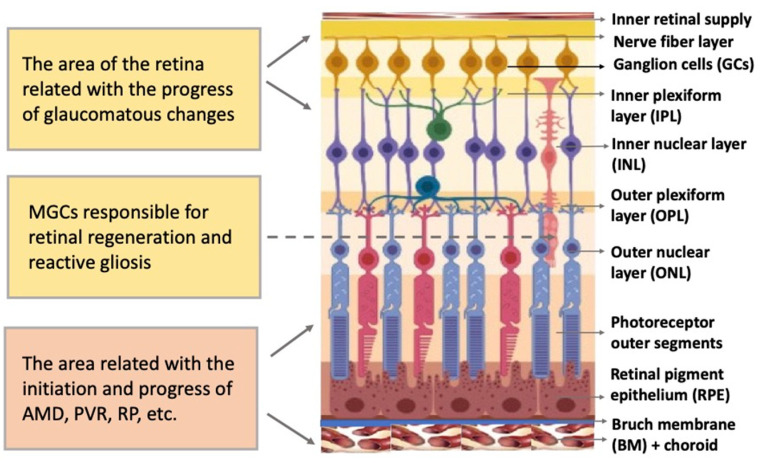
Structure of the retina and retinal compartments involved in degenerative diseases. MGCs—Müller glial cells, AMD—age-related macular degeneration, PVR—proliferative vitreoretinopathy, RP—retinitis pigmentosa.

The vertebrate retina is organized by a single plan, which, however, has specific morphological and functional features [22,23]. The retina is a neural, highly structured, stratified tissue where different types of neurons have strict localization, maintaining a stereotypic pattern of the NR (Figure 2). Functionally, it is a sensory tissue consisting of ordered layers whose cells interact with one another and neurons of other layers to provide light perception, receiving, and transmitting visual information. The retina is formed by six major NR cell types and RPE cells. NR cell populations are represented by photoreceptors (rods and cones), bipolars, and horizontal, amacrine, and ganglion cells. Müller glial cells (MGCs), microglia cells, astrocytes, and oligodendrocytes are integrated into the NR. The NR consists of three nuclear layers and two reticulate (plexiform) layers formed by fibers and synaptic contacts of neurons. The outer nuclear layer (ONL) is composed of bodies of photoreceptors, rods and cones. Their processes establish topological and functional connections with RPE. The inner nuclear layer (INL) contains bipolar, horizontal, and amacrine cells. INL interneurons are responsible for the visual signal transmission from photoreceptors to ganglion cells constituting the ganglion layer. The long processes of the ganglion cells compose the optic nerve that transmits information to the visual analyzer of the brain. The outer plexiform layer (OPL) is formed by fibers and synaptic contacts between photoreceptors and bipolar; the inner plexiform layer (IPL), by processes and synapses between bipolar and ganglion cells, as well as by horizontal processes forming connections between horizontal and amacrine cells. Bodies of the MGCs are located in the INL, extending their processes to the inner and outer limiting membranes of the NR, and are involved in their formation. In mammals and human the NR has two vascular supplies, the choroidal vasculature underlying RPE and the vessels of the inner retina (Figure 2). The blood supply to the inner retina is via the central retinal artery, whose branches radiate from the optic nerve head onto the inner retinal surface and then give rise to branches that penetrate into the retina through the INL, IPL, and OPL [24].

## 3. Brief Characteristics of the Major Degenerative Disorders of the Retina

**Figure 3:**
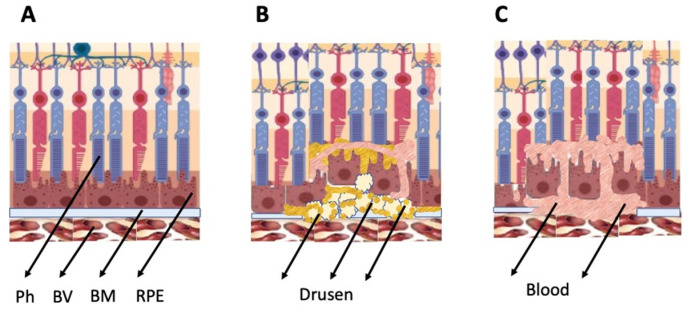
Schematic representation of AMD-related changes in the eye. (**A**)—normal eye; (**B**)—“dry” AMD; (**C**)—“wet” AMD. Ph—photoreceptors, BV—blood vessels, BM—Bruch membrane; RPE—retinal pigment epithelium.

As mentioned above, the general types of retinal degeneration include AMD, glaucoma, RP, and PVR. All of these disorders, except glaucoma, are caused by the loss of cells and cell–cell interactions in the functional light perception system, RPE and NR (Figure 2). AMD, affecting, according to approximate estimates, a quarter or more of the global population aged 65+, is accompanied by the loss of photoreceptors in the maculae region, where the light rays are focused on the retina. There are two forms of AMD: the “dry” (prevailing) and “wet” AMD [25]. With the dry AMD in an atrophic form, extracellular matrix molecules accumulate in the space outside the RPE, which causes the formation of so-called druses, consisting of fats, vitronectin, amyloid proteins, and inflammatory proteins, accumulated inside the RPE layer (Figure 3). These changes occur in RPE within the maculae region, causing partial cell death, layer disorganization, disruption of RPE functions, and para-inflammatory reaction, which inevitably results in the loss of photoreceptors [26,27]. The wet AMD, also referred to as neovascular (exudative) form, is manifested as the proliferation of a network of blood vessels lining the RPE choroidal membrane in the maculae region. Vessels become dysfunctional and leaky, with fluid and blood accumulating in the maculae region [28] (Figure 3). This causes disjunction of RPE apical processes and photoreceptors, while the connections between them are mandatory for light perception. AMD treatment is diverse, involving neurotrophic factors, growth factors, cell viability factors, and also oxidative stress-preventing factors [2,29,30,31]. With the wet AMD, vascular endothelial growth factor (VEGF) inhibitors and the photodynamic therapy are mainly used [32,33]. Despite efforts aimed at developing adequate therapy, the challenges associated with the treatment of AMD to preserve vision are still substantial. In this regard, the idea of cell replacement with the help of cell sources, including intrinsic ones, to regenerate RPE and photoreceptors becomes highly relevant. The feasibility of such an approach is discussed below.

PVR, often accompanying retinal rupture, is manifested as the withdrawal of RPE cells outside the layer, their epithelial–mesenchymal transition (EMT), transformation, and involvement in the epiretinal membrane (EM) formation [34,35,36]. Proliferative diabetic retinopathy [37] and subretinal fibrosis [38] are also known to be caused by mesenchymal transformation of RPE. In the treatment of this range of disorders, studies of mechanisms and methods for preventing the EMT of RPE cells and the EM formation with their involvement, which prevent normal functional connection between RPE and photoreceptors from being restored, are of particular importance [39].

Retinitis pigmentosa (RP) is characterized as a heterogeneous genetic disorder that leads to progressive devolution of the retina. This congenital disorder has a heterogeneous genetic origin. Approximately 100 genes are known whose mutations may result in RP [40]. It is often accompanied by the loss of peripheral and night vision. This is explained by the initial death of rods, as well as changes in the choroidal network, which, while progressing, leads to the degeneration of cones. Various approaches are currently being developed to slow down the progress of the disorder, including gene therapy, pharmacology, neuroprotection, electrical stimulation, retinal prostheses, and retinal transplantation [41]. Vitamin A preparations and other agents that improve retinal trophy are administered. Since the disorder affects the outer part of the eye—the choroidal coat, RPE, and photoreceptor cells—it would also be relevant to consider the feasibility of replacing damaged cells with genetically healthy ones.

Glaucoma is also one of the major retinal diseases leading to impairment of vision, often irreversible. Glaucoma represents a degenerative optic neuropathy characterized by the progressive degeneration of retinal ganglion cells and the retinal nerve fiber layer. Glaucomatous alterations, often associated with intraocular pressure increase, remain inconspicuous for a long time, but in the final stage, they lead to the death of bodies and axons of neurons, including those in the optic nerve head region [42]. The ocular hypertension and deleterious mechanical forces exerted at the back of the eye, at the level of the optic nerve head/optic disc, are the only modifiable risk factors associated with glaucoma that can be treated. The main approach to treatment is to reduce the intraocular pressure by administering prostaglandins and β-blockers [43,44]. Laser trabeculoplasty and surgery are also used to reduce the rate of disease progression [42]. However, as the death of ganglion cells increases, the necessity arises to replace them with poorly differentiated autogenous RRCSs capable of regenerating the ganglion layer and establishing correct connections with the INL and visual center neurons. 

## 4. Intrinsic Retinal Regeneration Cell Sources and Their Implication for Biomedicine of the Eye

Certain RRCS categories are described in brief in the subsections below. General information is provided in dedicated reviews [3,5,7,8,11,45] and in numerous specialized experimental works. The consideration of each RRCS type is accompanied by an attempt to analyze the prospects for using the accumulated knowledge about RRCSs in biomedicine of the eye.

### 4.1. Retinal Ciliary Zone Cells

RRCSs of this category are located on the extreme periphery of the retina, in the so-called corner of the eye (Figure 1). In vertebrates, the ciliary zone cells exhibit pronounced, to varying degrees, features of stemness/early progenitors. There are differences in the size of this cell population, decreasing in the series from fish to mammals. In fish and amphibians, persistent neurogenesis occurs in the poorly differentiated ciliary marginal zone (CMZ) that provides recruitment of cells to the growing retina [46,47]. The cells of this zone are also involved in the regeneration of the retina in case of damage [46,48,49,50]. 

CMZ cells of lower vertebrates have been studied as regards their internal molecular genetics and epigenetic profiles, and also functions of the signaling microenvironment. Expression of transcription factors (TFs) responsible for neurogenesis and regulatory signaling pathways that activate and/or block neurogenesis in the CMZ has also been investigated [3,51,52,53]. Eye field TFs in development belong to those whose expression is characteristic of CNS cells and causes manifestation of their neurogenic potential [54,55]. Their spectrum is encoded by members of the homeobox gene family: *Pax6* and *Rx*, *sine oculis*, *Six3*, and *Lhx*. Their list includes the genes *Chx10* (*vsx2* in fish), *Crx*, and *Pitx*, being also active at early stages of eye development [56,57].

Basic information about the CMZ has been collected through studies on the retina of fish [51,58,59] and amphibians [46,60] in development and regeneration. In these animals, the CMZ is composed of peripherally located stem cells and more centrally localized progenitors. The former are capable of continuous independent divisions; the latter divide only to a limited, regulated number [51,61,62,63]. The *Xenopus laevis* model has shown that the expression of genes encoding eye field TFs is characteristic of the most peripheral part of the CMZ. In *X. laevis,* Rx^+^ cells of the CMZ prior to metamorphosis are involved in retinal regeneration after partial incision; the *Rx* knockdown could impair regeneration by leading to a drastic reduction in proliferation [64]. The expression of *Rax* downstream genes was detected in the CMZ of *X. laevis* mature retina, which indicates the progenitor properties of these cells [65]. A study of gene expression in CMZ cells of mature caudate amphibians has also shown the progenitor characteristics of cells of this zone, which is involved, along with RPE, in epimorphic NR regeneration in these animals [49]. Expression of TFs Pax6, Otx2, Six3, and also Prox1 and Pitx2 was recorded for this zone in newts *Pl. waltl* [66,67,68]. In order to increase the neurogenic potential of the CMZ, attempts have been made to enhance the cell proliferation and differentiation in this zone using fish and amphibian models. This can be exemplified by the ability of CMZ cells to maintain a high level of proliferative activity, as has been reported for *dmbx1a* germline zebrafish mutants [69]. It has also been shown that the activation of the IGF receptor gene (*Igf1r*) in medaka causes a decrease in the cell cycle length and an increase in the production of differentiated neurons [70].

In reptiles, birds, and mammals, this retinal region is reduced, however, with few cells still retaining signs of a low level of differentiation and proliferation ability [50,71,72,73]. The CMZ in chicks before hatching is similar to that of adult fish and amphibians, but its contribution to retinal growth is small [74,75]. After hatching, the CMZ in birds does not disappear completely, and the neurogenesis remains extremely limited [76,77,78,79]. An attempt has been made to obtain neurons in vitro using cells from the ciliary margin of the chicken retina, whose neurogenic potential in vivo is very low [80]. Cultivation of cells isolated from the ciliary margin caused them to form so-called neurospheres. The neurosphere cells exhibited apicobasal polarity and acquired positional values along the radial and tangential axes close to those observed during NR development in vivo. When the neurospheres were placed in differentiation-promoting media, the cells acquired the phenotype of ganglion cells, forming long processes. In the latter, the key regulators of axon growth including, in particular, Eph/ephrin were identified [80]. The study showed for the first time the potential for reproduction of autologous, differentiated ganglion cells from the ciliary margin of the chicken retina in vitro. Though carried out on an animal model, the study provides a basis for attempts to reproduce human NR ganglion cells in vitro for transplantation purposes.

In mammals, the CMZ represents a very small number of cells localized in the area of transition from the retina to the ciliary body (CB) at the extreme periphery of the NR. In humans, it is a non-laminated part of the NR, also referred to as *ora serrata* [81]. Normally, these cells do not show proliferative activity and, as was previously suggested, mammals should lack any analogue of CMZ. However, a study of NR ciliary margin cells in mouse embryos revealed a limited neurogenesis with ganglion cell formation [82]. It is also known that cells of the marginal region of mammalian and human NR in early development express such TFs as Pax6, Chx10, Lhx2, Otx1, Prox1, Pitx1/2, retinol dehydrogenase Rdh10, and other markers, thus indicating a low level of differentiation of these cells [83,84,85]. In prenatal mice, cells of the NR marginal zone are positive towards BMP4, the Cyclin D2 proliferation factor, and also the TFs Msx1 and Zic1/2 [82,85,86]. There is evidence that in one of the adult mouse lines, cells of the NR marginal region retain the neurogenic potential and express *Atoh7*, a marker gene of neurogenesis [72]. The expression of *Atoh7* is assumed to be an autonomous response of the NR ciliary margin cells to apoptosis of ganglion cells. With the targeted elimination of part of the ganglion cells, these cells implement the neurogenesis potential, which results in the recruitment to the ganglion cell population [72]. There is also evidence of the proliferative activity of cells of the NR ciliary epithelium in rats with experimentally modeled RP [87]. Authors of this study explored the proliferation potential of ciliary marginal cells of Royal College of Surgeons (RCS) rats, an animal model for RP. A few Chx-10 and BrdU labeled cells were found, and their number significantly increased on day 30 and 60 of RCS postnatal development. 

These results suggest that the cells in the proliferating marginal region of the mammalian retina have the potential to regenerate the retina following its degeneration caused by RP [87].

Spontaneous migration of NR *ora serrata* cells, isolated in human explants and cultured in vitro, has recently been detected [88]. Co-expression of proliferation markers and Müller glial cell markers has also been observed in migrated cells. Proliferation of the NR ciliary epithelium cells with the subsequent acquisition of neuronal/terminal differentiation by them has been described for cases of retinal detachment and PVR in adult humans [89]. These observations suggested that the epidermal growth factor receptor (EGFR)-positive ciliary epithelium cells could form “neurosphere-like” structures and their differentiation was directed towards the neural and photoreceptor (but not glial) lineage formation. Therefore, in the adult human eye, the ciliary epithelium in a pathological retinal environment such as retinal detachment and PVR may provide a spontaneous source of donor cells for retinal transplantation [89].

These data indicate that the ciliary margin cells of the mammalian and human NR in embryonic development retain some features of progenitor cells. Under targeted experimental conditions or NR pathology in vivo, cells of the ciliary region are capable of proliferation and production of retinal cells. Understanding the molecular basis and searching for such conditions are among the promising objectives of regenerative biomedicine of the eye, aimed at reproducing ganglion, glial or other phenotypes of retinal cells to replace dysfunctional or lost ones.

In this regard, information about the external regulators of RRCS behavior in the CMZ is of particular importance. The major signaling regulatory molecules in the microenvironment of CMZ cells are IGF, Shh, Wnt, Notch, and glucagon [69]. In the NR development, CMZ cells, against the background of Notch activation and in its interaction with the TF Atoh7, are responsible for the production of bipolar, amacrine cells, and MGCs. A certain balance between Notch and Atoh7 is considered as a basis during maturation of the range of neurons produced by the CMZ in the postembryonic period [90]. In *X. laevis*, the Shh ligand, produced by ganglion cells, accelerates the cell cycle exit by suppressing cell proliferation in the CMZ. The effect of Shh signaling is exerted through the activation of cyclins, with the reduction in the length of the G_1_ and G_2_ phases of the cell cycle [91]. In chicks, overexpression of Shh in Pax6-positive CMZ cells induces expression of *Gli3/Gli1*, mediators of Shh signaling, which, in turn, enhances proliferation in the CMZ [92]. The factors activating proliferation in the CMZ are the Wnt ligand or expression of its effector genes found in mouse, chicken, frog, and fish [93,94,95,96]. In mouse, the activity of Wnt signaling is suppressed by the TFs Six3/Six6. In the ciliary marginal region of the retina, the *Six3/Six6* expression is reduced, which leads to up-regulation of the Wnt signaling pathway that supports the progenitor characteristics of cells [97]. Glucagon is also a factor regulating the proliferation of CMZ cells. In the chick NR, glucagon is normally produced by one of the populations of amacrine cells (bullwhip amacrines), extending their long processes to the CMZ and suppressing the proliferative activity with glucagon there [98]. 

There is information about the epigenome of CMZ cells obtained using animal models. Angileri and Gross [99] studied DNA methylation in CMZ cells by investigating the work of the Dnmt1 methyltransferase catalyst. The role of expression of this gene in the maintenance of progenitor properties by CMZ cells was determined using zebrafish mutants with knocked out *dnmt1*. The *dnmt1*-deficient fish (*dnmt1^−/−^)* showed a decrease not only in the number of cells in the CMZ, but also in their proliferative activity. The authors suggest that the *dnmt1*-related epigenetic modification in lower vertebrates is responsible for maintaining the poorly differentiated state of CMZ cells [99]. Histone acetylation plays a certain role in neuroprotection in the CMZ during neurogenesis. Histone deacetylase inhibitors (HDACi) can protect CMZ cells from death in mutant (*dying on edge*, *dye*) zebrafish that have mass cell death in this zone [100]. To date, all of the hypothesized variety of epigenetic mechanisms responsible for and regulating the progenitor status of cells and neurogenesis in the CMZ during the NR development and regeneration requires in-depth study.

The obtained data on CMZ cells were considered by Miles and Tropepe [69] in association with microphthalmia. This genetically inherited disorder is related to a deficiency in progenitor cell proliferation in this region of the eye. Myopia, a well-known vision condition, is also associated with the growth and shape of the eye that, in turn, are related with the cell behavior in the retinal growth region [101].

Taking into account the collected information about the extremely limited potentials of the mammalian NR ciliary zone, any natural formation of new neurons or RPE cells in this zone in humans is improbable. Nevertheless, the extensive information about the genetic properties of ciliary region cells and the regulatory molecules of their progenitor state, proliferation, and neurogenesis can potentially be applied in biomedicine of the eye in the future. Furthermore, this knowledge contributes to the development of therapies for not only retinal diseases directly associated with developmental CMZ disorders, but also diseases requiring cell replacement. Reproduction of ganglion cells in vitro, as shown in the above-cited studies by Fiore et al. [80] on a bird model, can be developed using material from embryonic and adult rodents. Cells with progenitor properties and Müller glial cells can be produced by cultivating fragments of tissue from the same area of the retina that are obtained during eye surgery in humans and usually discarded [88].

### 4.2. Ciliary Body Cells

As mentioned above, in adult mammals, the region similar to the CMZ is extremely reduced by the number of cells and does not normally exhibit any regenerative abilities [102]. A region, close in localization but not analogous to the CMZ of lower vertebrates, in adult mammals and humans is represented by the ciliary body (CB) (Figure 1). The CB in the mammalian eye has two cell layers and muscles. The outer pigmented layer is a continuation of the RPE; the inner, non-pigmented layer, a continuation of the NR. The cells constituting the CB have a specialization different from that of RPE and NR neurons. They produce components of vitreous fluid and are involved in visual accommodation [103,104]. However, in case of damage to the NR leading to the loss of ganglion and amacrine cells, cell proliferation is known to be activated in the non-pigmented CB layer [6,102,105,106,107]. In adult mice, some CB cells re-enter the cell cycle and change their phenotype, expressing TF Chx10 and also marker proteins of bipolar and photoreceptors as a response to the optic nerve transection causing the death of ganglion cells [105]. Expression of retinal progenitor genes was observed in the adult mouse CB after intraocular injections of regulators of Rho GTPase activity [108]. The injections enhanced the co-expression of TFs Pax6 and Chx10, but showed no effect on proliferation in the CB. The inactivation of Rho GTPases conversely increased the proliferation of CB cells, including those exposed to growth factors. The authors suggest that the approach to and understanding of the ways of regulation of CB cell proliferation and differentiation can be used to replace dead ganglion and photoreceptor cells with CB cells [108].

Numerous in vitro studies have shown that human, primate, pig, rodent, and chicken CB cells can express Nes, Mitf, Pax6, Six3, Rx, Chx10, and FGF2, which are markers of stem cells and retinal progenitors [109,110,111,112,113]. In a number of studies [109,114,115], cell aggregates (neurospheres) were obtained from dissociated mouse and human CB cells. As the authors assumed, these aggregates were formed by multipotent cells. The cells within the neurospheres proliferated, were nestin (Nes) and Pax6 positive, and simultaneously expressed Claudin-1, a marker protein of epithelial cells. Cells that expressed protein markers of retinal neurons, in particular, photoreceptors, were found in rare cases. After further investigating the issue, Cicero et al. [116] concluded that CB cells are not truly stem cells, as they retain certain features of pigmented epithelia. Such results and the low production of retinal precursors by descendants of CB cells in neurospheres in vitro have reduced interest in CB as a potential cell source to replace dying NR neurons [117]. However, attempts are still being made to increase the production of neurons from CB in vitro by manipulating cultivation conditions [118,119,120]. In a study on cultures of ciliary/CB epithelium cells of postnatal pigs [121], expression of pluripotency marker genes *Klf4*, *Sox2*, and *cMyc* was detected in a suspension of cells derived from neurospheres. After the latter were dissociated and labeled with CM-DiI vital dye, the cells were injected subretinally into the eyes of adult mice. After transplantation, the cells acquired a mixed phenotype, showing features of retinal neurons and RPE cells. Some of them were incorporated in the RPE, where multilayered RPE65^+^ cell loci were observed. Another portion of the cells (5–10%) expressed marker proteins of retinal neurons: recoverin, protein kinase C, and calbindin [121]. These results directly indicate the expediency of continuing the studies on CB cell potencies to be used for cell replacement in the treatment of retinal degeneration.

Little is known about the external regulators of CB cell reprogramming in vitro. As early studies [122,123] showed, when Notch signaling is disrupted, the formation of neurospheres from CB cells is blocked. Notch has recently been discovered to play a role in the development of CB, where combinations of Notch family proteins are necessary for morphogenesis, differentiation, and the establishment of CB functions [124]. Thus, Notch 3 in cooperation with Notch 2, regulating the adhesion factor Nectin1, is responsible for the CB morphogenesis. Pang et al. [124] suggest that Notch regulation may underlie the progression of glaucoma. Signaling including the tyrosine kinase receptor, c-Kit transmembrane protein and its ligand, stem cell factor, are also assumed to be involved in the regulation of reprogramming of CB cells forming neurospheres in vitro. These components are capable of supporting not only the proliferative activity of cells in CB-derived spheres, but also their differentiation in the retinal direction [122,123]. 

In a study of CB-derived neurosphere cells in vitro, some epigenetic regulators of retinal cell differentiation were identified. For this, Jasty and Krishnakumar [125] analyzed DNA methylation and histone methylation-H3K4me3 and H3K27me3 in a CB-derived lineage committed progenitor to terminally differentiated cells isolated from the CB of human cadaveric eyes. The authors detected bivalent modifications involved in the process of differentiation of stem/progenitor cells into neural and glial cells [125].

Thus, the mammalian, including human, CB contains cells capable of acquiring retinal differentiation. These are few in number and their initial differentiation in vivo is stabilized, which is consistent with the functional destination in the CB. However, studies of molecular genetics and epigenetic profiles, as well as the potential of reactivation to proliferation and genesis of cells with signs of retinal differentiation revealed under experimental conditions, still indicate CB cells as potential candidates for application in cell technologies. In addition to the accumulated knowledge that can be used to obtain an in vitro cell resource for replacing RPE and dead NR neurons, the data on the biology of CB cells and its regulation can contribute to the development of glaucoma therapy [42].

### 4.3. Retinal Pigment Epithelium Cells

In adult vertebrates and humans, RPE is a monolayer of pigmented, epithelial, and specialized cells. The RPE is oriented towards the NR with its apical side; on the basal side, it is limited by the Bruch’s membrane and the vascular membrane referred to as choroid (Figure 1 and Figure 2). The RPE is multifunctional: apart from transferring substances from the choroid to the NR, it protects against oxidative stress, produces growth factors, and metabolizes vitamin A derivatives. A major RPE function is phagocytosis of the outer segments of photoreceptors, their digestion by lysosomes, and retinoid metabolism, i.e., providing the processes required for light perception [126,127,128,129].

Among the well-known RRCSs, RPE has been studied to a greater extent, including the possible implications for practical use. Damage to the RPE layer and its cells, and also disturbance of their relationship with photoreceptors, are the causes of most degenerative diseases of the retina. In this regard, and taking into account the common origin of RPE and NR in the development of the eye and the possibility of their mutual conversion [39], RPE has been extensively studied using animal models and in humans. Studies have been conducted in various directions: cell functions, differentiation of RPE and its changes during regeneration, and congenital and acquired eye pathologies; RPE behavior in vitro and under transplantation conditions has also been investigated [127,130,131,132,133,134,135,136].

A study on a model of zebrafish larvae and adults has shown that RPE regeneration is possible even in the case of significant damage to the layer. To develop an RPE injury model, a transgenic line (*rpe65a*:nfsB-eGFP) of fish was used [137]. The regeneration of the RPE layer occurred through proliferation of cells adjacent to the area of damage, compensating for the loss of the layer. Based on transgenic and mutant zebrafish lines, pharmacological manipulations, and transcriptomic and morphological analyses, the authors investigated the immune response mediating the regeneration of the RPE layer. They found that RPE cells express the immune response genes, in particular, interleukin 34, and that macro- and microglial cells are necessary to maintain this immune response [138]. 

RPE of caudate amphibians (Urodela) is a classic example showing the potential for natural regeneration of the RPE layer along with its reprogramming into NR cells in mature animals in vivo. Even in the case where the original NR is removed in newts, the RPE becomes a source of a new, functioning NR and, simultaneously, restores its own layer [133,139,140,141,142,143]. During NR regeneration, RPE cells leave the layer, lose the initial features of specialization, proliferate, and form an intermediate population of cells with neuroblast properties. After six or seven cycles of cell divisions increasing the population of the NR regenerate, neuroblasts acquire phenotypes of retinal neurons and glial cells and begin to function. The initial number of cells in the RPE layer is restored through the proliferation of cells retained in it. The process of newt RPE reprogramming, the NR anlage formation, and its differentiation are regulated by TFs, signaling molecules, and epigenetic controllers. After the disconnection of RPE and NR, the genes of the immune response and proto-oncogenes (*c-fos*, *c-myc*, and *c-jun*) are activated first [144]. It was also found that the daughter cells of RPE at the beginning of retinectomy-induced proliferation express the pluripotency genes *c-myc*, *Klf4*, and *Sox2* and, along with them, developmental *Mitf* and *Pax6* [145] and the neural stem cell marker Musashi-1 (Msi-1) [146]. Expression of genes characteristic of eye development (*Pax6, Prox1, Six3, Pitx1,* and *Pitx2*) along with tissue-specific *RPE65* and *Otx2* has also been revealed [66,68,142,147,148]. The patterns of expression of both marker and regulatory molecules in NR development and regeneration are largely similar. In an experiment by Casco-Robles [149], RPE cells acquired mesenchymal-like phenotype, and NR regeneration was blocked. This was caused by the knockout of the *Pax6* gene in larval newt *Cynops pyrrhogaster.* Leaving the RPE layer, these cells formed aggregates showing the expression of myofibroblast proteins (a-SMA, Vim, and N-Cad). These data are important for understanding the role of the Pax6 gene in the mesenchymal transformation of RPE cells that occurs in cases of PVR and retinal fibrosis in humans [34,35,36]. 

Studies of regulation of RPE cell differentiation by the microenvironment also provide clues to the directed reprogramming of these cells. Among the signaling cascades controlling NR regeneration in Urodela and zebrafish, special attention is paid to the Fgf, Bmp, Wnt, Shh, and Notch signaling pathways [150,151,152,153]. The key role of FGF2 in NR regeneration in newts (as in other animals) has been identified [154,155,156]. It has been found that FGF2 is not the primary trigger of RPE reprogramming, but is required for regulating *pax6* gene expression at the onset of the process and then for increasing the proliferation and entering the differentiation of RPE-derived cells [152,157,158]. As for other RRCSs of the eye, the neural conversion of RPE in salamanders depends on Notch signaling. According to [150] and [151], the introduction of the DAPT blocker induces premature maturation of neurons in the NR regenerate. The mechanism of epigenetic regulation of the RPE reprogramming into NR cells in salamanders has not been studied, but the permissive factors of the epigenome are assumed to be the expression of pioneer TFs (opening repressed chromatin domains) and demethylation of regulatory elements of photoreceptor genes [159].

As in newts, RPE potencies to regenerate NR through reprogramming have been found in the frog *X. laevis* [160,161]. The up-regulation of the “developmental” *pax6* and *rax* has been shown for the reprogramming of cells derived from RPE of larval *X. laevis*. Knockdown of the *rax* gene inhibits the formation of specific cell types in the NR regenerate [162,163]. The expression of the *rx* gene is necessary for the formation of NR regenerate in pre-metamorphic *X. laevis* [64,164]. The key external regulator of RPE conversion in *X. laevis* is FGF2 promoting the proliferation [161,165]. Inhibition of the FGF2-dependent MAPK signaling pathway reduces the size of the NR cell population forming the regenerate [161]. Matrix metalloproteases show positive regulation of the NR regeneration from RPE cells and are, in turn, regulated by the inflammatory cytokines IL-1β and TNF-α [166]. The model of NR regeneration from RPE in *X. laevis* is reported to allow a wide range of applications of molecular genetics methods to identify the mechanisms triggering the activity of regeneration-associated genes [167]. 

In chicks, NR regeneration from RPE cells occurs at early ontogeny stages (up to E4–E4.5) [168,169]. A fragment of C3a complement is considered to be an inducer of the NR regeneration process in chicks. This short-lived polypeptide activates STAT3, enhancing the action of IL-6, IL-8, and TNF factors, which leads to up-regulation of the Wnt2b signaling genes and expression of the *Six3* and *Sox2* genes characteristic of retinal progenitor cells [170]. A study on an embryonic chick model (stage E4) [171] has revealed nuclear β-catenin as an obstacle to the entry of RPE cells into the cell cycle. In the presence of FGF2, its expression was lost, thus, indicating the inactivation of nuclear β-catenin as one of the conditions for NR regeneration from RPE cells in chicks [171]. The involvement of Shh [172], BMP, and Wnt signaling cascades [173] in NR regeneration has been reported for birds as well. It is also known that during the formation of the RPE and NR domains in the optic cup of the chick embryo, a low concentration of BMP leads to the RPE to NR conversion, whereas a high one conversely leads to the NR to RPE changeover [173]. Along with BMP, Wnt signaling plays an important role in such transformations. The authors suggest that the control of these two key signaling pathways can provide the basis of a protocol for the production of RPE and NR cells for cell replacement. In the study by Tangeman et al. [174], laser capture microdissection was used to isolate RNA from intact RPE, transiently reprogrammed RPE (t-rRPE) 6 h post-retinectomy, and reprogrammed RPE (rRPE) 6 h post-retinectomy with FGF2 treatment. RNA sequencing of individual cells (scRNA-seq) and a comparative analysis of transcriptomes were carried out. At an early stage of RPE conversion, up-regulation of damage-associated genes and repression of genes responsible for the entry and progression of the cell cycle were detected. In the presence of FGF2, on the contrary, the level of expression of MAPK activation genes was high, which confirms the assumption that the FGF2/MAPK signaling cascade is the main driver of RPE conversion in embryonic chickens [174]. An attempt was made to understand the mechanisms of the EMT strategy of mammalian RPE cells in PVR or fibrosis using the same animal model. It turned out that the expression of EMT-associated genes was suppressed at the onset of the RPE conversion, but later activated. The expression of EMT-associated genes (*SNAI1*, *TGFBR2*, *ELK3*, *SMAD3*, and *TGFB3*), presumably dependent on extracellular matrix, is modulated during these events. Thus, parallel studies of the expression of genes responsible for the neuronal conversion of RPE cells and the genes responsible for EMT can answer the question as to why RPE of avian embryos avoids mesenchymal transformation [174].

Variations in the phenotype and proliferative activity of RRCSs occur with the involvement of the changing epigenetic landscape that modifies the transcription program. Using an embryonic chick model, Luz-Madrigal et al. [175] carried out TAB-seq and ChIP-seq to study the process of NR regeneration from RPE. RPE cells were studied prior to retinectomy and at various RPE reprogramming stages. The data indicated a significant rearrangement of the DNA methylation pattern. Regions of differential methylation of gene promoter sites, associated with chromatin organization and FGF2 production, were identified. The study highlights the implication of DNA demethylation in RPE cells for overcoming epigenetic barriers to NR regeneration from RPE in mammals and humans [175]. Thus, the competence of RPE cells in amphibians and birds to be reprogrammed into retinal cells and the mechanisms of their implementation are based, like in other animals, on the extracellular and intracellular features of molecular regulation of cell behavior. Understanding of these facts, taking into account the conservatism of the main tools to regulate the differentiation and proliferation of RPE cells in animals and humans, is of fundamental importance for the development of biotechnological approaches aimed at regeneration of damaged human retina.

In mammals and humans, RPE layer regeneration is extremely limited: only very small damages can be repaired through expansion of neighboring cells. An exception to this rule is mutant MRL/MPJ mice, in which repair of minor damage to RPE is possible on the periphery of the layer [176]. A study by Kampik et al. [177], carried out also on genetically modified mice, showed the probability of partial RPE regeneration with the lack of cell transformation into neural or other phenotypes. According to our observations during experiments with organotypic 3D cultivation of the posterior sector of the rat eye, the RPE layer under conditions of partial death of its cells still continued to maintain the epithelial structure for a long time. This occurs through the increase in the size of cells and their stretching over the layer; however, such RPE cells cannot establish a normal functional connection with photoreceptors, simultaneously undergoing death [178]. The models of in vivo RPE damage in mice showed an increase in the size of RPE cells and also in the proportion of multinucleated cells [179,180]. When damaged or disconnected from the NR, the RPE is known to produce a wide range of factors, including PEDF, BDNF, and CNTF [181,182]. These RPE responses are of reparative nature and aimed at maintaining the viability of cells and their integration with the NR. The RPE in mammals, including humans, is not capable of regeneration, despite the fact it has rare cell divisions detected on the periphery of the layer [134,183,184]. 

**Figure 4:**
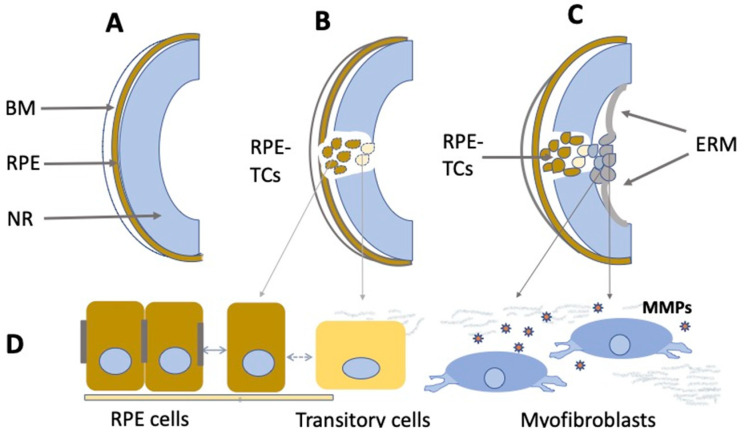
Schematic illustration of PVR development. (**A**)—Normal eye. BM—Bruch’s membrane, RPE—retinal pigment epithelium, NR—neural retina. (**B**)—RPE at the beginning of the epithelial– mesenchymal transition. RPE-TCs—RPE-derived transforming cells. (**C**)—Epiretinal membrane (ERM) formation. (**D**)—Morphological changes of RPE cells during the epithelial–mesenchymal transition. MMPs—Matrix metalloproteases.

In vivo proliferative activity and depigmentation of RPE cells are known for a number of pathological conditions of the retina. Proliferation of RPE cells accompanies the above-mentioned vitreoretinopathy (PVR). In the first stage of the disease, often associated with NR detachment, a portion of RPE cells lose their pigment and epithelial properties, leave the layer, migrate beyond the NR, and proliferate (Figure 4). These processes are accompanied by the death of RPE cells or their conversion into myofibroblasts. During migration, while exposed to the surrounding conditions, RPE cells proliferate, initiate the synthesis of extracellular matrix components, and are involved in epiretinal membrane (EM) formation [185,186,187,188,189]. The formation of EM and its contraction along with NR are responsible for the clinical manifestation of PVR. The key event of PVR is the epithelial–mesenchymal transition (EMT) of RPE cells [35,36,190,191]. Note that the EMT occurs not only in PVR, but also in the case of the RPE attempting to recover and restore the layer after laser-induced damage [192], subretinal fibrosis [193,194], or proliferative diabetic retinopathy [37]. The EMT is manifested as the loss of the polarity of RPE cells, destruction of their contacts, and detachment from the Bruch’s membrane. These events are accompanied by the cytoskeleton reorganization and acquisition of a mesenchymal phenotype [195,196]. A reorganization of cell–cell contacts occurs when the expression patterns of cadherins and associated catenins change [197,198]. Smooth muscle alpha actin (α-SMA) is an intracellular protein, a marker of transitory epithelial–mesenchymal differentiation that provides mobility of RPE cells. Vimentin is responsible for stabilizing the structure of RPE-derived cells during migration [199]. As EMT progresses, the pattern of cytokeratin expression also is altered, and extracellular matrix proteins, including collagen and fibronectin, are deposited [198]. 

Variations in the expression of functionally and structurally significant genes under the control of TFs, epigenetic factors, and external regulatory signaling systems constitute the molecular genetic basis for the EMT of RPE cells [200,201]. The role of the FOXM1 proto-oncogene as a regulator of EMT and RPE cell proliferation has been identified [202,203]. The expression pattern of TFs belonging to the Snail and Slug, ZEB1/2, TWIST, GSC superfamilies [204,205] and other TFs usually accompanying EMT in fibrous or onco-transformation is known to undergo alteration with the EMT [206,207]. The TFs that control the key EMT mechanism—the initiation of expression of E-cadherins—play a major regulatory role. This range of TFs is represented by Oct-1, hepatocyte nuclear factor 1 (HNF-1), such TFs as GATA-1, SMAD3, and TFE, interferon regulatory factor-1 (IRF), etc. [205,208].

Micro RNAs (miRNAs) are also involved in the regulation of gene expression in the PVR process [209,210]. Maps of the associated epigenetic and transcriptional changes in human RPE with simulated EMT, compared to the normal ones, have been reconstructed. Active enhancers and TFs associated with actively transcribed genes have been identified in studies of the RPE epigenome and transcriptome in normal conditions and after treatment with key positive EMT regulators, TGF-β1 and TNF-α. In parallel, nicotinamide (NAM) has been found to be able to suppress EMT-associated key transcription events in human RPE cells [211]. The external mediators of the EMT process in PVR are growth factors and inflammatory factors. The process is initiated by TGF-β [212] and develops with the involvement of TNF-α, PDGF, EGF, FGF, VEGF, CTGF, IGF2, IL-1a,β, IL-2,3,6,8, adhesion factor ICAM-1, and other signaling [205,213,214,215,216]. An increase in the TGF-β level was observed in vitreous bodies of patients with PVR, which correlated with the severity of the disease [217]. TNF-α enhances the expression of genes associated with apoptosis and cell motility in RPE [218]. Both factors synergistically activate the EMT program in adult RPE cells. Modulation of RhoA/Rho-kinase, Smad, or MAPK signaling is considered among the strategies for suppressing signaling pathways involved in the RPE pathology [212,219]. Exposure to the above-mentioned NAM can enhance the epithelial phenotype of RPE and prevent the EMT. For this reason, NAM is considered an agent for preventive treatment of RPE/retinal pathologies [211,220]. Other approaches involve blocking the expression of one of the TGF-β receptors such as, in particular, activin receptor-like kinase 5 (ALK5), which facilitates suppression of the EM formation [221,222]. The causes and progression of PVR, resulting from genetic aberrations that alter the behavior of RPE cells, are the subject of further molecular research to provide sufficient data for biomedicine of the eye. 

It seems that the impossibility of restoring the RPE layer and regenerating NR from it should discourage researchers from considering RPE as a potential RRCS in mammals including humans. However, data on RPE cells’ behavior in vitro not only revive these hopes, but also contribute to understanding of the RPE-associated retinal diseases. The data from in vitro experiments clearly indicate the plasticity of the mammalian RPE phenotype and the potential of de- and re-differentiation, and also of reprogramming into other retinal cell phenotypes. Rodent and human RPE cells exposed to morphogens and growth factors in the culture medium lose their original properties and proliferate [223,224,225,226]. Adult rat RPE cells in vitro express marker proteins of neural progenitors: Nes, Musashi1, doublecortin, and β-III tubulin [224]. As was shown on mouse embryos, the neural conversion of RPE is blocked by activin, one of the TGFβ superfamily proteins [227]. A series of studies of isolated human RPE cells and cell lineages in vitro revealed a decrease in the level of specific differentiation and expression of RPE65 along with the activation of the progenitor cell marker genes *OCT4*, *NANOG*, *KLF4*, *OTX2*, *PAX6*, and *NES* [225,228,229]. In some cases, the expression of pan-neural markers, tyrosine hydroxylase and neurofilament proteins was recorded [225]. The introduction of FGF2 into the medium caused RPE cells to exhibit proneural properties [230]. Analyzing the behavior of RPE cells depending on their cultivation conditions, Burke [231] found a relationship of the phenotype modulations of RPE cells with the Wnt/β-catenin signaling. There is evidence of the role of the highly conserved Hippo signaling in this process [232]. It has also been reported that mouse RPE cells forming neurospheres in vitro represent a population whose cells are capable of returning to the original phenotype or acquiring photoreceptor differentiation. When subretinally transplanted into mice with retinal degeneration, RPE-derived neurosphere cells could integrate into the RPE and NR, thereby delaying retinal degeneration. This finding clearly shows the perspectives for the use of mammalian RPE cells to replace dying cells in NR diseases [232]. 

Study of the mesenchymal conversion of human RPE cells and its regulation in vitro is of certain interest for biomedicine in the context of PVR therapy. A study by Salero et al. [233] showed that human RPE cells in vitro can reproduce cells expressing markers of mesenchyme-derived cells: muscle, adipo-, osteo- and chondrogenic cells. This occurs where the components stimulating the manifestation of these differentiations are added to the medium. The Wnt signaling pathway plays a significant role in the regulation of mesenchymal differentiation in the ARPE-19 cell line in vitro [234]. Based on the behavior of mammalian and human RPE cells during in vitro cultivation, some authors [232,233,235,236] consider them (or a small number of them in the RPE layer) stem cells. A recent study by Pandey et al. [237], based on the results of scRNA sequencing, revealed the molecular features of heterogeneity in the adult murine RPE. Approximately 1–2% of the total number of cells exhibited the hallmarks of stem and/or progenitor cells. The authors suggest that RPE stem cells may also exist in the human tissue. 

Compared to the data on the potential of the neural and mesenchymal conversion of the human RPE, the information on ways to keep RPE cells from reprogramming and stabilize their initial phenotype through signaling molecules in vitro is no less important. Studies of transcriptomes of such RPE cells maintaining initial differentiation in vitro indicate that they retain properties close to native ones [238,239,240,241].

Thus, the results of the studies on RPE as a source of retinal regeneration/degeneration in animals and humans in vivo and the behavior of mammalian RPE cells in vitro make an undoubtedly substantial contribution to biomedicine of the eye. This contribution is determined by the observed ability of RPE to produce retinal neurons and by the identification of external and internal mechanisms of RPE cell phenotype regulation. The former allows obtaining autologous cells in vitro to replace dying cells in the NR. The latter makes it possible to control the behavior of RPE cells to prevent their death and undesirable phenotypic transformations, design methods for the treatment of retinal diseases, and also study the molecular genetic basis of such diseases.

### 4.4. Müller Glial Cells

Müller glial cells (MGCs) are well-known and widely studied as latent RRCSs (see reviews [242,243,244,245,246]). According to data obtained recently, MGCs that have undergone age-related changes can still be stimulated to regenerate cells lost after acute NR damage in aged zebrafish [247]. MGCs are very promising for regenerating cell losses in the NR [8,245,248,249]. 

**Figure 5:**
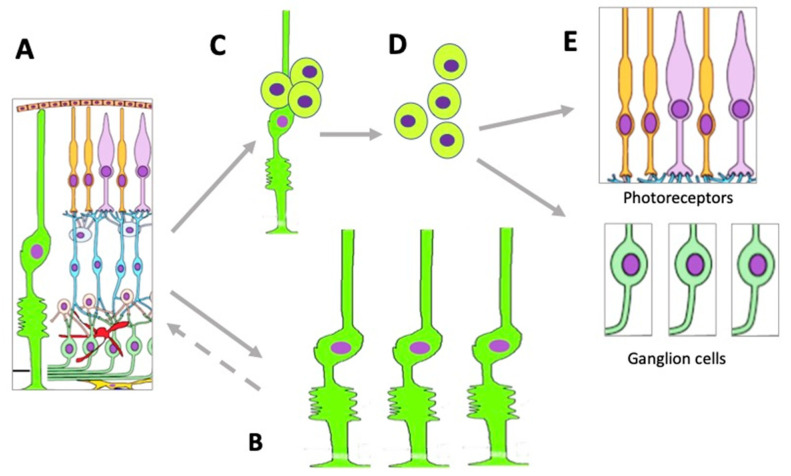
Changes occurring in the MGC population under conditions of retinal damage. (**A**)—MGCs in the structure of normal retina; (**B**)—MGC hypertrophy and proliferation in conditions of reactive gliosis; (**C**)—MGC reprogramming and proliferation during retinal regeneration in vivo and after directed stimulation in vitro; (**D**)—MGC-derived retinal cell precursors emerging during retinal regeneration in vivo and after directed stimulation in vitro; (**E**)—retinal neurons formed from MGC-derived retinal cell precursors. See more detailed description in the text.

MGC bodies are located radially in the INL, extending long processes to the outer and inner limiting membranes of the NR (Figure 2). The Müller glia is a cell population specialized in performing a wide range of functions, including neurotrophic and structural ones, and also maintaining synaptic connections with NR neurons. It is involved in both NR cleaning and light perception [250]. Furthermore, extensive evidence indicates that MGCs are a population that can exhibit the properties of neural progenitor cells. If NR is damaged, they re-express TFs (six3, pax6, rx1, olig2, and vsx2) characteristic of NR progenitors and immature macroglial cells [251]. In the case of surgical excision of zebrafish NR, MGCs proliferate and produce retinal progenitors capable of differentiating into photoreceptors (cones) and interneurons [252]. After thermal or light-induced damage to the fish NR causing loss of photoreceptors, MGCs re-enter the cell cycle and subsequently up-regulate the expression of stem and progenitor cell-specific proteins [51,253,254]. During the life-long growth of the eye in fish, MGCs maintain the rod photoreceptor lineage, and in case of regeneration, they are able to produce precursors for photoreceptors and ganglion cells [242]. Emerging evidence shows that inflammation plays an essential role in the multi-step process of retinal regeneration [255]. The zebrafish model, with its extensive experimental manipulation capabilities, has been accepted for the study of MGC reprogramming in order to stimulate MGC conversion in mammals [256] (Figure 5).

In birds after hatching, MGCs exposed to the growth factors IGF and FGF2 also demonstrate regenerative responses: they proliferate and express “developmental” TFs [77,257,258]. The transcriptome of MGCs in postnatal mammals shows homology with that of dividing NR progenitors during ontogeny. After the first postnatal week in mice, 68% of the genes specifically expressed in MGCs match those of proliferating progenitors, and only 14% are associated with photoreceptor differentiation [259]. Differentiated MGCs of the mouse retina are characterized by the expression of genes necessary for proliferation [260]. In the normal adult NR in vivo, MGCs express CD117 and CD44, which are marker proteins associated with stem cells, and, simultaneously, vimentin, a marker of glial differentiation [261]. It is found also that adult human MGCs express markers of neural progenitors including SOX2, PAX6, CHX10 and NOTCH, and become spontaneously immortalized in vitro [262]. Authors suggest that MGCs constitute a potential resource of retinal neurons for transplantation studies. 

Simultaneously, mammalian retinal MGCs respond to damage by reactive gliosis, manifested as up-regulation of stress proteins, proliferation, hypertrophy of MGCs, and strengthening of the radial glia differentiation traits [263] (Figure 5). Such a behavior of MGCs is characteristic of many pathological conditions of the NR accompanied by the death of neurons. Reactivation of MGCs is aimed at protecting the NR tissue from destruction and supporting its functions through the release of antioxidants and neurotrophic factors [264]. The extent of neuronal death determines the proliferative activity of MGCs in mice, while the factors secreted by neurons provoke MGCs to activate the EGFR-ERK pathway necessary for proliferation. This, in turn, suggests that cell death-associated signaling pathways may be considered a therapeutic target to prevent proliferative gliosis in NR degeneration [265].

MGCs of the mature NR proliferate, not only in conditions of NR pathology in vivo, but also when cultivated in vitro [266,267]. Components of the EGF, FGF, Notch, Wnt, and Shh signaling pathways, when exogenously added to the culture medium, can provoke mammalian MGCs to exhibit the properties of retinal progenitors [257,268,269,270,271,272]. Data obtained on human MGCs also indicate the genetic traits characteristic of RRCSs, i.e., a combination of genes associated with both NR progenitors and functional specialization genes [45]. MGCs obtained from the NR of cadaveric human eye samples and cultured in vitro under exposure to growth factors showed a decrease in the level of differentiation, acquiring the phenotype of proneural precursors [273,274]. Taking these features into consideration, it seems relevant to search for regulators that allow or block gliosis and reprogramming of mammalian, including human, MGCs.

MGCs are also related to the diabetic retinopathy, in which they dysregulate the neuronal function and produce proangiogenic and pro-inflammatory factors, thus, creating an environment facilitating neuronal dysfunction [275]. In this regard, restoration and maintenance of normal MGC functions in the case of diabetic retinopathy is considered as a key method for the treatment of this disease. Additionally, approaches are known that enhance the functioning of MGCs by stimulating the beta-adrenergic pathway [276].

To date, along with the already studied systemic and regulatory factors of the MGCs (CNTF, GDNF, FGF2, IGF, Notch-Delta, Wnt/b-catenin, etc.) [7,9,51,277], new ones such as miRNAs have also become known [278,279]. Overexpression of miR-25 (or let-7) in antagonism with miR-124 induces the expression of the proneural TF Ascl1, providing reprogramming of mature MGCs into NR cells in mice [280]. miR-9/9* and miR-124 act as negative regulators of the Sox2-Ascl1a/Atoh7-Lin-28 pathway, inhibiting proliferation, and as activators of the TLX-ONECUT signaling, stimulating MGC differentiation into retinal neurons [281]. In addition to their role in the regulation of MGC behavior, miRNAs are involved in the progression of glaucoma and ocular pressure regulation. In the very-near future, a study of this issue and, in particular, the delivery of miRNAs in order to develop miRNA-based glaucoma therapies is required [282]. 

Epigenetic features of MGCs are also subject to consideration. Many authors indicate the similarity in molecular genetics between mature MGCs and late progenitors for bipolars and photoreceptors (rods) [259,260,283,284]. Data by Dvoriantchikova et al. [284], characterizing the epigenetic profile of MGCs, confirm this. The results of ChIP-seq suggest that the obstacles to NR regeneration by MGCs in mammals are explained by the state of chromatin suppressing the work of genes responsible for the late stages of MGC reprogramming, in particular, the specification of retinal neuron types. This problem may be addressed by means of pioneering TFs and DNA demethylase activity [284]. There is evidence of changes in the DNA methylation/demethylation [285]. MGCs are assumed to maintain the plasticity of differentiation that allows them to be reprogrammed and, as a result, regenerate dead NR cells by inducing additional epigenetic modifications of the genome [285,286]. Jorstad et al. [286], using a histone deacetylase inhibitor and providing overexpression of the key regulator of MGC reprogramming, *Ascl1* gene [287], managed to stimulate neurogenesis in the adult mouse MGC population destroyed by a neurotoxin at the stage of their development, when the natural manifestation of neurogenic potential had already been blocked. The descendant cells of MGCs, pretreated this way, expressed the marker proteins of interneurons, formed synapses with pre-existing NR neurons, and responded to a light stimulus [286].

The results of a comparative study of the genetic network controlling the regenerative activity of MGCs in fish, birds, and mice have been published recently [288]. The differences in gene expression and chromatin availability in MGCs in response to various stimuli in these animals were assessed by RNA-seq and ATAC-seq. Evolutionarily conserved genetic networks with specific features and that control MGCs were identified in a resting state, in a reactive state, and during neurogenesis. In all animals, NR damage induces reactivation and reprogramming of MGCs; however, in the mouse regulation network, including nuclear factors 1 (NF1), cells return to a resting state. The deletion of the NF1-encoding genes allows mouse MGCs to complete the final stages of cell conversion, i.e., produce retinal neurons. Thus, NF1 is another key factor in the regulation network involved in suppression of neurogenesis in the mammalian MGC population [288].

An unusual study by Bonilla-Pons et al. [289] has been conducted on an organotypic culture of NR using cell fusion technology. As a result, it turns out that, as in mice [290], human MGCs can fuse with human mesenchymal stem cells, giving rise to hybrid cells. Such hybrids are capable of producing retinal neurons, and their pretreatment with the activator Wnt/β-catenin increases their differentiation into neuronal-like cells, providing the ability of the hybrid descendants to migrate, engraft, and manifest neural, proto-electrophysiological properties. The authors [289] suggest that this technique of cell fusion-mediated MGC reprogramming and neurogenesis in the human NR is another potential therapeutic approach to cell regeneration for the treatment of human NR diseases. It should also be mentioned that the presence of stem cells is known to enrich the cell environment in trophic molecules and self-renewal factors and to enhance cell homing and survival [291,292]. In turn, the enriched environment exhibits neuroprotective properties, as has been evidenced, in particular, by an induced model of ocular hypertension [293].

The new information that human surgical NR explants having ciliary epithelium in their structure contain cells with the retinal precursor cell properties seems encouraging [88]. These cells from surgical explants obtained through vitrectomy for the management of rhegmatogenous detachment, when placed under cultivation conditions without growth factors, are able to migrate, proliferate, and express progenitor markers: Pax6, Sox2, CRX, nestin, p27^kip1^, and also marker proteins of MGCs (GFAP, GS). The authors note that these cells obtained from patients can be a source of MGCs and other retinal progenitors [88].

## 5. Conclusions

The results of in vivo and in vitro studies indicate that intrinsic retinal regeneration cell sources (RRCSs), which include RPE, CB, MG, and the NR ciliary region, have intrinsic genetic features that determine their potencies for retinal neuron production. These potencies are implemented, to varying degrees, from complete or partial retinal regeneration by RRCSs in fish, amphibians, and bird embryos to the manifestation of certain progenitor properties, proliferation, and change of the RRCS phenotype in mammals. In the latter, as well as in humans, such regenerative responses are most frequently found under conditions of directed induction in vitro. Identification of RRCSs and mechanisms of regulation of their behavior have long been conducted in various studies on animal models and humans. The studies have shown that the mechanisms regulating the manifestation/inhibition of regenerative responses of RRCSs in animals and humans are common. In general, these include external signaling, changing transcription patterns, and the epigenetic landscape. The pathways of conversion through proliferation and acquisition of a progenitor state that cells can leave by acquiring a new differentiation of one or more retinal phenotypes are also common for latent RRCSs (RPE, CB, and MGCs). In the progenitor state phase, RRCSs express “developmental” TFs and multipotency genes that are controlled by intracellular mechanisms of genome regulation. These mechanisms, in turn, depend on a wide range of external effects such as signaling molecules (growth factors, inflammation, viability, cell death, hormones, etc.). Modulation of immune responses during degenerative processes in the NR can also affect the course of NR regeneration. 

The major degenerative diseases of the retina are associated with changes, death, and loss of function by the RPE and photoreceptors. Diseases of this kind include AMD, PVR, and RP. Glaucoma is caused by the loss of ganglion cells, while reactive gliosis, accompanying many NR pathologies, is caused by cell hypertrophy and an increase in the MGC population. To translate data to biomedicine of the eye, studies of RRCSs are being conducted in two main areas. The first is a search for technologies to provide replacement of dead/degenerating NR cells with healthy ones obtained from endogenous RRCSs. Designing the methods to promote the production of autologous retinal cells de novo under in vivo or in vitro conditions is an important alternative to the use of stem cells for these purposes. The use of exogenous stem cells in ophthalmology is widely studied currently. Nevertheless, despite marked technological advances, the risks of mutations, tumor growth, undesirable benign transformations of these cells, etc. have been recorded. Obtaining the required cell types from stem cells with integration of new cells into the cell ensemble and, at last, the issue of immune rejection, still pose serious challenges. The natural retinal regeneration process achieved through the use of RRCSs may be more successful for therapy, including the facilitated integration of endogenous cells and proper synaptic targeting. However, the issue of competition between the use of endogenous RRCSs and stem cells is not addressed here. As can be seen from the examples discussed above, these methods can be coupled and complement each other.

There is another important implication of the knowledge of RRCSs in biomedicine of the eye. The information about the molecular-genetic status of RRCSs and the molecular mechanisms regulating their behavior makes it possible to prevent RRCS-associated pathological processes, such as AMD, PVR, reactive gliosis, and glaucoma. In the case of such pathologies, the knowledge of the RRCS control mechanisms contributes to the development of therapy targeted at inhibiting the active manifestation of RRCS-related disorders. Despite the evident successes in the study of the mechanisms of internal and external regulation of RRCS behavior, further research in this field is still required to elucidate, in particular, the fate of RRCS-derived cells during transplantation and their integration in the damaged mammalian retina. It is obvious that the direct use of RRCSs is not a close prospect today. However, as discussed in the review, with the application of techniques for intracellular and external regulation of RRCS behavior in animal models, the desired goals of cell replacement and/or correction of degenerative changes in the NR can be achieved.

## Data Availability

Not applicable.

## References

[1] Varma R., Vajaranant T.S., Burkemper B., Wu S., Torres M., Hsu C., Choudhury F., McKean-Codwin P. (2016). Visual impairment and blindness in adults in the United States: Demographic and geographic variations from 2015 to 2050. JAMA Ophthalmol..

[2] Pardue M.T., Allen R.S. (2018). Neuroprotective strategies for retinal disease. Prog. Retin. Eye Res..

[3] Moshiri A., Close J., Reh T.A. (2004). Retinal stem cells and regeneration. Int. J. Dev. Biol..

[4] Lamba D., Karl M., Reh T. (2008). Neural regeneration and cell replacement: A view from the eye. Cell Stem Cell.

[5] Karl M.O., Reh T.A. (2010). Regenerative medicine for retinal diseases: Activating endogenous repair mechanisms. Trends Mol. Med..

[6] Wohl S.G., Schmeer C.W., Isenmann S. (2012). Neurogenic potential of stem/progenitor-like cells in the adult mammalian eye. Prog. Retin. Eye Res..

[7] Yu H., Vu T.H., Cho K.S., Guo C., Chen D.F. (2014). Mobilizing endogenous stem cells for retinal repair. Transl. Res..

[8] Madelaine R., Mourrain P. (2017). Endogenous retinal neural stem cell reprogramming for neuronal regeneration. Neural. Reg. Res..

[9] Grigoryan E.N. (2018). Molecular Factors of the Maintenance and Activation of the Juvenile Phenotype of Cellular Sources for Eye Tissue Regeneration. Biochemistry.

[10] Grigoryan E.N. (2018). Endogenous cell sources for eye retina regeneration in vertebrate animals and human. Russ. J. Dev. Biol..

[11] Aladdad A.M., Kador K.E. (2019). Adult Stem Cells, Tools for Repairing the Retina. Curr. Ophthalmol. Rep..

[12] Ramsden C.M., Powner M.B., Carr A.J., Smart M.J., da Cruz L., Coffey P.J. (2013). Stem cells in retinal regeneration: Past, present and future. Development.

[13] Sowden J.C. (2014). ESC-derived retinal pigmented epithelial cell transplants in patients: So far, so good. Cell Stem Cell.

[14] Jeon S., Oh I.H. (2015). Regeneration of the retina: Toward stem cell therapy for degenerative retinal diseases. BMB Rep..

[15] Nazari H., Zhang L., Zhu D., Chader G.J., Falabella P., Stefanini F., Rowland T., Clegg D.O., Kashani A.H., Hinton D.R. (2015). Stem cell-based therapies for age-related macular degeneration: The promises and the challenges. Prog. Retin. Eye Res..

[16] Singh M.S., Park S.S., Albini T.A., Canto-Soler M.V., Klassen H., MacLaren R.E., Takahashi M., Nagiel A., Schwartz S.D., Bharti K. (2020). Retinal stem cell transplantation: Balancing safety and potential. Prog. Retin. Eye Res..

[17] German O.L., Vallese-Maurizi H., Soto T.B., Rotstein N.P., Politi L.E. (2021). Retina stem cells, hopes and obstacles. World J. Stem Cells.

[18] Schwartz S.D., Regillo C.D., Lam B.L., Eliott D., Rosenfeld P.J., Gregori N.Z., Hubschman J.P., Davis J.L., Heilwell G., Spirn M. (2015). Human embryonic stem cell-derived retinal pigment epithelium in patients with age-related macular degeneration and Stargardt‘s macular dystrophy: Follow-up of two open-label phase 1/2 studies. Lancet.

[19] Shim S.H., Kim G., Lee R.D. (2017). Survival of Transplanted Human Embryonic Stem Cell-Derived Retinal Pigment Epithelial Cells in a Human Recipient for 22 Months. JAMA Ophthalmol..

[20] Da Cruz L., Fynes K., Georgiadis O., Kerby J., Luo Y.H., Ahmado A., Vernon A., Daniels J.T., Nommiste B., Hasan S.M. (2018). Phase 1 clinical study of an embryonic stem cell-derived retinal pigment epithelium patch in age-related macular degeneration. Nat. Biotechnol..

[21] Han I.C., Bohrer L., Gibson-Corley K., Wiley L.A., Shrestha A., Harman B.E., Jiao C., Sohn E.H., Wendland R., Allen B.N. (2022). Biocompatibility of Human Induced Pluripotent Stem Cell-Derived Retinal Progenitor Cell Grafts in Immunocompromised Rats. Cell Transplant..

[22] Masland R.H. (2012). The Neuronal Organization of the Retina. Neuron.

[23] Hoon M., Okawa H., Della Santina L., Wong R.O. (2014). Functional architecture of the retina: Development and disease. Prog. Retin. Eye Res..

[24] Alm A., Hart W.M. (1992). Ocular circulation. Adler’s Physiology of the Eye: Clinical Application.

[25] Fernandes A.-R., Zielińska A., Sanchez-Lopez E., dos Santos T., Garcia M.L., Silva A.M., Karczewski J., Souto E.B. (2022). Exudative versus Nonexudative Age-Related Macular Degeneration: Physiopathology and Treatment Options. Int. J. Mol. Sci..

[26] Al-Hussaini H., Schneiders M., Lundh P., Jeffery G. (2009). Drusen are associated with local and distant disruptions to human retinal pigment epithelium cells. Exp. Eye Res..

[27] Ding X., Patel M., Chan C.C. (2009). Molecular pathology of age-related macular degeneration. Prog. Retin. Eye Res..

[28] Chirco K., Sohn E., Stone E., Tucker B., Mullins R. (2017). Structural and molecular changes in the aging choroid: Implications for age-related macular degeneration. Eye.

[29] Novikova Y.P., Gancharova O.S., Eichler O.V., Philippov P.P., Grigoryan E.N. (2014). Preventive and Therapeutic Effects of SkQ1-Containing Visomitin Eye Drops against Light-Induced Retinal Degeneration. Biochemistry.

[30] Jaffe G.J., Ciulla T.A., Ciardella A.P., Devin F., Dugel P.U., Eandi C.M., Masonson H., Monés J., Pearlman J.A., Quaranta-El Maftouhi M. (2017). Dual antagonism of PDGF and VEGF in neovascular age-related macular degeneration: A phase IIb, multicenter, randomized controlled trial. Ophthalmology.

[31] Ammar M.J., Hsu J., Chiang A., Ho A.C., Regillo C.D. (2020). Age-related macular degeneration therapy: A review. Curr. Opin. Ophthalmol..

[32] Han J.W., Choi J., Kim Y.S., Kim J., Brinkmann R., Lyu J., Park T.K. (2018). Comparison of the neuroinflammatory responses to selective retina therapy and continuous-wave laser photocoagulation in mouse eyes. Graefe’s Arch. Clin. Exp. Ophthalmol..

[33] Khanna S., Komati R., Eichenbaum D.A., Hariprasad I., Ciulla T.A., Hariprasad S.M. (2019). Current and upcoming anti-VEGF therapies and dosing strategies for the treatment of neovascular AMD: A comparative review. BMJ Open Ophthalmol..

[34] Morescalchi F., Duse S., Gambicorti E., Romano M.R., Costagliola C., Semeraro F. (2013). Proliferative Vitreoretinopathy after Eye Injuries: An Overexpression of Growth Factors and Cytokines Leading to a Retinal Keloid. Mediat. Inflamm..

[35] Idrees S., Sridhar J., Kuriyan A.E. (2019). Proliferative Vitreoretinopathy: A Review. Int. Ophthalmol. Clin..

[36] Zou H., Shan C., Ma L., Liu J., Yang N., Zhao J. (2020). Polarity and epithelial-mesenchymal transition of retinal pigment epithelial cells in proliferative vitreoretinopathy. Peer J..

[37] Abu El-Asrar A.M., Midena E., Al-Shabrawey M., Mohammad G. (2013). New Developments in the Pathophysiology and Management of Diabetic Retinopathy. J. Diabetes Res..

[38] Lopez P.F., Yan Q., Kohen L., Rao N.A., Spee C., Black J., Oganesian A. (1995). Retinal pigment epithelial wound healing in vivo. Arch Ophthalmol..

[39] Grigoryan E.N. (2022). Pigment Epithelia of the Eye: Cell-Type Conversion in Regeneration and Disease. Life.

[40] Hartong D.T., Berson E.L., Dryja T.P. (2006). Retinitis pigmentosa. Lancet.

[41] Pach J., Gekeler F. (2013). Therapeutic approaches for retinitis pigmentosa. Klin. Monbl. Augenheilkd..

[42] Weinreb R.N., Aung T., Medeiros F.A. (2014). The pathophysiology and treatment of glaucoma: A review. JAMA.

[43] Cholkar K., Trinh H.M., Pal D., Mitra A.K. (2015). Discovery of novel inhibitors for the treatment of glaucoma. Expert Opin. Drug Discov..

[44] Prum B.E., Herndon L.W., Moroi S.E., Mansberger S.L., Stein J.D., Lim M.C., Rosenberg L.F., Gedde S.J., Williams R.D. (2016). Primary Angle Closure Preferred Practice Pattern^®^ Guidelines. Ophthalmology.

[45] Grigoryan E.N. (2020). Potential Endogenous Cell Sources for Retinal Regeneration in Vertebrates and Humans: Progenitor Traits and Specialization. Biomedicines.

[46] Harris W.A., Perron M. (1998). Molecular recapitulation: The growth of the vertebrate retina. Int. J. Dev. Biol..

[47] Hitchcock P.F., Raymond P.A. (2004). The teleost retina as a model for developmental and regeneration biology. Zebrafish.

[48] Johns P.R. (1977). Growth of the adult goldfish eye. III. Source of the new retinal cells. J. Comp. Neurol..

[49] Mitashov V.I., Panova I.G., Koussoulakos S. (2004). Transdifferentiation potencies of ciliary and pigment epithelium cells of lower vertebrates and mammals. Russ. J. Dev. Biol..

[50] Fischer A.J., Bosse J.L., El-Hodiri H.M. (2013). The ciliary marginal zone (CMZ) in development and regeneration of the vertebrate eye. Exp. Eye Res..

[51] Raymond P., Barthel L.K., Bernardos R.L., Perkowski J.J. (2006). Molecular characterization of retinal stem cells and their niches in adult zebrafish. BMC Dev. Biol..

[52] Borday C., Cabochette P., Parain K., Mazurier N., Janssens S., Tran H.T., Sekkali B., Bronchain O., Vleminckx K., Locker M. (2012). Antagonistic crossregulation between Wnt and Hedgehog signaling pathways control post-embryonic retinal proliferation. Development.

[53] Cerveny K.L., Varga M., Wilson S.W. (2012). Continued growth and circuit building in the anamniote visual system. Dev. Neurobiol..

[54] Zuber M.E., Gestri G., Viczian A.S., Barsacchi G., Harris W.A. (2003). Specification of the vertebrate eye by a network of eye field transcription factors. Development.

[55] Viczian A.S., Solessio E.C., Lyou Y., Zuber M.E. (2009). Generation of functional eyes from pluripotent cells. PLoS Biol..

[56] Wang J.C., Harris W.A. (2005). The role of combinational coding by homeodomain and bhlh transcription factors in retinal cell fate specification. Dev. Biol..

[57] Wehman A.M., Staub W., Meyers J.R., Raymond P.A., Baier H. (2005). Genetic dissection of the zebrafish retinal stem-cell compartment. Dev. Biol..

[58] Otteson D.C., Hitchcock P.F. (2003). Stem cells in the teleost retina: Persistent neurogenesis and injury-induced regeneration. Vis. Res..

[59] Stenkamp D.L. (2007). Neurogenesis in the Fish Retina. Int. Rev. Cytol..

[60] Perron M., Kanekar S., Vetter M.L., Harris W.A. (1998). The genetic sequence of retinal development in the ciliary margin of the *Xenopus* eye. Dev. Biol..

[61] Centanin L., Ander J.J., Hoeckendorf B., Lust K., Kellner T., Kraemer I., Urbany C., Hasel E., Harris W.A., Simons B.D. (2014). Exclusive multipotency and preferential asymmetric divisions in post-embryonic neural stem cells of the fish retina. Development.

[62] Shi D., Tavhelidse T., Thumberger T., Wittbrodt J., Greb T. (2017). Bifacial stem cell niches in fish and plants. Curr. Opin. Genet. Dev..

[63] Wan Y., Almeida A.D., Rulands S., Chalour N., Muresan L., Wu Y., Simons B.D., He J., Harris W.A. (2016). The ciliary marginal zone of the zebrafish retina: Clonal and time-lapse analysis of a continuously growing tissue. Development.

[64] Martinez-De Luna R.I., Kelly L.E., El-Hodiri H.M. (2011). The Retinal Homeobox (Rx) gene is necessary for retinal regeneration. Dev. Biol..

[65] Pan Y., Kelly L.E., El-Hodiri H.M. (2018). Identification of retinal homeobox (rax) gene-dependent genes by a microarray approach: The DNA endoglycosylase neil3 is a major downstream component of the rax genetic pathway. Dev. Dyn..

[66] Markitantova Y.V., Makar’ev E.O., Smirnova Y.A., Zinov’eva R.D., Mitashov V.I. (2004). Analysis of the expression pattern of regulatory genes *Pax6*, *Prox1*, and *Six3* during regeneration of eye structures in the newt. Biol. Bull..

[67] Avdonin P.P., Markitantova Y.V., Zinov’eva R.D., Mitashov V.I. (2008). Expression of regulatory genes *Pax6*, *Otx2*, *Six3*, and *FGF2* during newt retina regeneration. Biol. Bull..

[68] Avdonin P.P., Grigoryan E.N., Markitantova Y.V. (2010). Transcriptional factor Pitx2: Localization during triton retina regeneration. Biol. Bull..

[69] Miles A., Tropepe V. (2021). Retinal Stem Cell ‘Retirement Plans’: Growth, Regulation and Species Adaptations in the Retinal Ciliary Marginal Zone. Int. J. Mol. Sci..

[70] Becker C., Lust K., Wittbrodt J. (2021). Igf signaling couples retina growth with body growth by modulating progenitor cell division. Development.

[71] Ghai K., Stanke J.J., Fischer A.J. (2008). Patterning of the circumferential marginal zone of progenitors in the chicken retina. Brain Res..

[72] Kiyama T., Li H., Gupta M., Lin Y.P., Chuang A.Z., Otteson D.C., Wang S.W. (2012). Distinct neurogenic potential in the retinal margin and the *pars plana* of mammalian eye. J. Neurosci..

[73] Todd L., Suarez L., Squires N., Zelinka C.P., Gribbins K., Fischer A.J. (2016). Comparative analysis of glucagonergic cells, glia and the circumferential marginal zone in the reptilian retina. J. Comp. Neurol..

[74] Prada C., Puga J., Perez-Mendez L., López A.R., Ramírez G. (1991). Spatial and temporal patterns of neurogenesis in the chick retina. Eur. J. Neurosci..

[75] Fischer A.J. (2005). Neural regeneration in the chick retina. Prog. Retin. Eye Res..

[76] Fisher A.J., Reh T.A. (2000). Identification of a proliferating marginal zone of retinal progenitors in postnatal chickens. Dev. Biol..

[77] Fischer A.J., Dierks B.D., Reh T.A. (2002). Exogenous growth factors induce the production of ganglion cells at the retinal margin. Development.

[78] Kubota R., Hokoc J.N., Moshiri A., McGuire C., Reh. T.A. (2002). A comparative study of neurogenesis in the retinal ciliary marginal zone of homeothermic vertebrates. Brain Res. Dev. Brain Res..

[79] Amato M.A., Arnault E., Perron M. (2004). Retinal stem cells in vertebrates: Parallels and divergences. Int. J. Dev. Biol..

[80] Fiore L., Carreño C.O., Medori M., Spelzini G., Sanchez V., Scicolone G. (2022). NeurospheRes. obtained from the ciliary margin of the chicken eye possess positional values and retinal ganglion cells differentiated from them respond to EphA/ephrin-A system. Exp. Eye Res..

[81] Bhatia B.L., Singhal S., Lawrence J.M., Khaw P.T., Limb G.A. (2009). Distribution of Muller stem cells within the neural retina: Evidence for the existence of a ciliary margin-like zone in the adult human eye. Exp. Eye Res..

[82] Marcucci F., Murcia-Belmonte V., Coca Y., Ferreiro-Galve S., Kuwajima N., Khalid S., Ross M.E., Mason C., Herrera E. (2016). The ciliary margin zone of the mammalian retina generates retinal ganglion cells. Cell Rep..

[83] Martinez-Morales J.R., Signore M., Acampora D., Simeone A., Bovolenta P. (2001). Otx genes are required for tissue specification in the developing eye. Development.

[84] Horsford D.J., Nguyen M.T., Sellar G.C., Kothary R., Arnheiter H., McInnes R.R. (2005). Chx10 repression of Mitf is required for the maintenance of mammalian neuroretinal identity. Development.

[85] Kuwahara A., Ozone C., Nakano T., Saito K., Eiraku M., Sasai Y. (2015). Generation of a ciliary margin-like stem cell niche from self-organizing human retinal tissue. Nat. Commun..

[86] Belanger M.C., Robert B., Cayouette M. (2017). Msx1-Positive progenitors in the retinal ciliary margin give rise to both neural and non-neural progenies in mammals. Dev. Cell.

[87] Jian Q., Xu H., Xie H., Tian C., Zhao T., Yin Z.Q. (2009). Activation of retinal stem cells in the proliferating marginal region of RCS rats during development of retinitis pigmentosa. Neurosci. Lett..

[88] Too L.K., Shen W., Mammo Z., Osaadon P., Gillies M.C., Simunovic M.P. (2021). Surgical retinal explants as a source of retinal progenitor cells. Retina.

[89] Ducournau Y., Boscher C., Adelman R.A., Guillaubey C., Schmidt-Morand D., Mosnier J.F., Ducournau D. (2012). Proliferation of the ciliary epithelium with retinal neuronal and photoreceptor cell differentiation in human eyes with retinal detachment and proliferative vitreoretinopathy. Graefe’s Arch. Clin. Exp. Ophthalmol..

[90] Saturnino A.P., Lust K., Wittbrodt J. (2018). Notch signaling patterns retinal composition by regulating atoh7 during post-embryonic growth. Development.

[91] Locker M., Agathocleous M., Amato M.A., Parain K., Harris W.A., Perron M. (2006). Hedgehog signaling and the retina: Insights into the mechanisms controlling the proliferative properties of neural precursors. Genes Dev..

[92] Moshiri A., McGuire C.R., Reh T.A. (2005). Sonic hedgehog regulates proliferation of the retinal ciliary marginal zone in posthatch chicks. Dev. Dyn..

[93] Liu H., Thurig S., Mohamed O., Dufort D., Wallace V.A. (2006). Mapping canonical Wnt signaling in the developing and adult retina. Investig. Ophthalmol. Vis. Sci..

[94] Denayer T., Locker M., Borday C., Deroo T., Janssens S., Hecht A., van Roy F., Perron M., Vleminckx K. (2008). Canonical Wnt signaling controls proliferation of retinal stem/progenitor cells in postembryonic xenopus eyes. Stem Cells.

[95] Kubo F., Nakagawa S. (2009). Hairy1 acts as a node downstream of Wnt signaling to maintain retinal stem cell-like progenitor cells in the chick ciliary marginal zone. Development.

[96] Meyers J.R., Hu L., Moses A., Kaboli K., Papandrea A., Raymond P.A. (2012). β-catenin/Wnt signaling controls progenitor fate in the developing and regenerating zebrafish retina. Neural. Dev..

[97] Diacou R., Zhao Y., Zheng D., Cvekl A., Liu W. (2018). Six3 and Six6 Are Jointly Required for the Maintenance of Multipotent Retinal Progenitors through Both Positive and Negative Regulation. Cell Rep..

[98] Fischer A.J., Omar G., Walton N.A., Verrill T.A., Unson C.G. (2005). Glucagon-expressing neurons within the retina regulate the proliferation of neural progenitors in the circumferential marginal zone of the avian eye. J. Neurosci..

[99] Angileri K.M., Gross J.M. (2020). dnmt1 function is required to maintain retinal stem cells within the ciliary marginal zone of the zebrafish eye. Sci. Rep..

[100] Daly C., Shine L., Heffernan T., Deeti S., Reynolds A.L., O’Connor J.J., Dillon E.T., Duffy D.J., Kolch W., Cagney G. (2017). A brain-derived neurotrophic factor mimetic is sufficient to restore cone photoreceptor visual function in an inherited blindness model. Sci. Rep..

[101] Wallman J., Winawer J. (2004). Homeostasis of eye growth and the question of myopia. Neuron.

[102] Karl M.O., Hayes S., Nelson B.R., Tan K., Buckingham B., Reh T.A. (2008). Stimulation of neural regeneration in the mouse retina. Proc. Natl. Acad. Sci. USA.

[103] Napier H.R., Kidson S.H. (2007). Molecular events in early development of the ciliary body: A question of folding. Exp. Eye Res..

[104] McDougal D.H., Gamlin P.D. (2015). Autonomic control of the eye. Comp. Physiol..

[105] Nickerson P.E., Emsley J.G., Myers T., Clarke D.B. (2007). Proliferation and expression of progenitor and mature retinal phenotypes in the adult mammalian ciliary body after retinal ganglion cell injury. Investig. Ophthalmol. Vis. Sci..

[106] Ooto S., Akagi T., Kageyama R., Akita J., Mandai M., Honda Y., Takahashi M. (2004). Potential for neural regeneration after neurotoxic injury in the adult mammalian retina. Proc. Natl. Acad. Sci. USA.

[107] Wohl S.G., Schmeer C.W., Kretz A., Witte O.W., Isenmann S. (2009). Optic nerve lesion increases cell proliferation and nestin expression in the adult mouse eye in vivo. Exp. Neurol..

[108] Del Debbio C.B., Santos M.F., Yan C.Y., Ahmad I., Hamassaki D.E. (2014). Rho GTPases control ciliary epithelium cells proliferation and progenitor profile induction in vivo. Investig. Ophthalmol. Vis. Sci..

[109] Ahmad I., Tang L., Pham H. (2000). Identification of neural progenitors in the adult mammalian eye. Biochem. Biophys. Res. Commun..

[110] Das A.V., James J., Rahnenfuhrer J., Thoreson W.B., Bhattacharya S., Zhao X., Ahmad I. (2005). Retinal properties and potential of the adult mammalian ciliary epithelium stem cells. Vis. Res..

[111] Lord-Grignon J., Abdouh M., Bernier G. (2006). Identification of genes expressed in retinal progenitor/stem cell colonies isolated from the ocular ciliary body of adult mice. Gene Expr. Patterns.

[112] MacNeil A., Pearson R.A., MacLaren R.E., Smith A.J., Sowden J.C., Ali R.R. (2007). Comparative analysis of progenitor cells isolated from the iris, pars plana, and ciliary body of the adult porcine eye. Stem Cells.

[113] Martinez-Navarrete G.C., Angulo A., Martin-Nieto J., Cuenca N. (2008). Gradual morphogenesis of retinal neurons in the peripheral retinal margin of adult monkeys and humans. J. Comp. Neurol..

[114] Tropepe V., Coles B.L., Chiasson B.J., Horsford D.J., Elia A.J., McInnes R.R., van der Kooy D. (2000). Retinal stem cells in the adult mammalian eye. Science.

[115] Coles B.L., Angenieux B., Inoue T., del Rio-Tsonis K., Spence J.R., McInnes R.R., Arsenijevic Y., van der Kooy D. (2004). Facile isolation and the characterization of human retinal stem cells. Proc. Natl. Acad. Sci. USA.

[116] Cicero S.A., Johnson D., Reyntjens S., Frase S., Connell S., Chow L.M., Baker S.J., Sorrentino B.P., Dyer M.A. (2009). Cells previously identified as retinal stem cells are pigmented ciliary epithelial cells. Proc. Natl. Acad. Sci. USA.

[117] Gualdoni S., Baron M., Lakowski J., Decembrini S., Smith A.J., Pearson R.A., Ali R.R., Sowden J.C. (2010). Adult ciliary epithelial cells, previously identified as retinal stem cells with potential for retinal repair, fail to differentiate into new rod photoreceptors. Stem Cells.

[118] Inoue T., Coles B.L., Dorval K., Bremner R., Bessho Y., Kageyama R., Hino S.l., Matsuoka M., Craft C.M., McInnes R.R. (2010). Maximizing functional photoreceptor differentiation from adult human retinal stem cells. Stem Cells.

[119] Ballios B.J., Clarke L., Coles B.L., Shoichet M.S., van der Kooy D. (2012). The adult retinal stem cell is a rare cell in the ciliary epithelium whose progeny can differentiate into photoreceptors. Biol. Open.

[120] Fernández-Nogales M., Murcia-Belmonte V., Chen H.Y., Herrera E. (2019). The peripheral eye: A neurogenic area with potential to treat retinal pathologies?. Prog. Retin. Eye Res..

[121] Guduric-Fuchs J., Chen W., Price H., Archer D.B., Cogliati T. (2011). RPE and neuronal differentiation of allotransplantated porcine ciliary epithelium-derived cells. Mol. Vis..

[122] Ahmad I., Das A.V., James J., Bhattacharya S., Zhao X. (2004). Neural stem cells in the mammalian eye: Types and regulation. Semin. Cell Dev. Biol..

[123] Das A.V., James J., Zhao X., Rahnenführer J., Ahmad I. (2004). Identification of c-Kit receptor as a regulator of adult neural stem cells in the mammalian eye: Interactions with Notch signaling. Dev. Biol..

[124] Pang J., Le L., Zhou Y., Tu R., Hou Q., Tsuchiya D., Thomas N., Wang Y., Yu Z., Alexander R. (2021). NOTCH Signaling Controls Ciliary Body Morphogenesis and Secretion by Directly Regulating Nectin Protein Expression. Cell Rep..

[125] Jasty S., Krishnakumar S. (2016). Profiling of DNA and histone methylation reveals epigenetic-based regulation of gene expression during retinal differentiation of stem/progenitor cells isolated from the ciliary pigment epithelium of human cadaveric eyes. Brain Res..

[126] Strauss O. (2005). The Retinal Pigment Epithelium in Visual Function. Physiol. Rev..

[127] Sparrow J.R., Hicks D., Hamel C.P. (2010). The Retinal Pigment Epithelium in Health and Disease. Curr. Mol. Med..

[128] Fuhrmann S., Zou C.J., Levine E.M. (2014). Retinal pigment epithelium development, plasticity, and tissue homeostasis. Exp. Eye Res..

[129] Lakkaraju A., Umapathy A., Tan L.X., Daniele L., Philp N.J., Boesze-Battaglia K., Williams D.S. (2020). The cell biology of the retinal pigment epithelium. Prog. Retin. Eye Res..

[130] Koster C., Wever K.E., Wagstaff P.E., van den Hirk K.T., Hooijmans C.R., Bergen A.A. (2020). A Systematic Review on Transplantation Studies of the Retinal Pigment Epithelium in Animal Models. Int. J. Mol. Sci..

[131] Caceres P.S., Rodriguez-Boulan E. (2020). Retinal Pigment Epithelium Polarity in Health and Blinding Diseases. Curr. Opin. Cell Biol..

[132] Markitantova Y., Simirskii V. (2020). Inherited Eye Diseases with Retinal Manifestations through the Eyes of Homeobox Genes. Int. J. Mol. Sci..

[133] Grigoryan E.N., Markitantova Y.V. (2021). Molecular Strategies for Transdifferentiation of Retinal Pigment Epithelial Cells in Amphibians and Mammals In Vivo. Russ. J. Dev. Biol..

[134] George S., Lu F., Rao M., Leach L., Gross J.M. (2021). The retinal pigment epithelium: Development, injury responses, and regenerative potential in mammalian and non-mammalian systems. Prog. Retin. Eye Res..

[135] Rizzolo L.J., Nasonkin I.O., Adelman R.A. (2022). Retinal Cell Transplantation, Biomaterials, and In Vitro Models for Developing Next-generation Therapies of Age-related Macular Degeneration. Stem Cells Transl. Med..

[136] Grigoryan E.N. (2022). Self-Organization of the Retina during Eye Development, Retinal Regeneration In Vivo, and in Retinal 3D Organoids In Vitro. Biomedicines.

[137] Hanovice N.J., Leach L.L., Slater K., Gabriel A.E., Romanovicz D., Shao E., Collery R., Burton E.A., Lathrop K.L., Link B.A. (2019). Regeneration of the zebrafish retinal pigment epithelium after widespread genetic ablation. PLoS Genet..

[138] Leach L.L., Hanovice N.J., George S.M., Gabriel A.E., Gross J.M. (2021). The immune response is a critical regulator of zebrafish retinal pigment epithelium regeneration. Proc. Natl. Acad. Sci. USA.

[139] Keefe J.R. (1973). An analysis of urodelean retinal regeneration. I–IV. J. Exp. Zool..

[140] Mitashov V.I. (1996). Mechanisms of retina regeneration in vertebrates. Int. J. Dev. Biol..

[141] Mitashov V.I. (1997). Retinal regeneration in amphibians. Int. J. Dev. Biol..

[142] Chiba C., Mitashov V.I., Chiba C. (2007). Cellular and molecular events in the adult newt retinal regeneration. Strategies for Retinal Tissue Repair and Regeneration in Vertebrates: From Fish to Human.

[143] Yasumuro H., Sakurai K., Toyama F., Maruo F., Chiba C. (2017). Implications of a Multi-Step Trigger of Retinal Regeneration in the Adult Newt. Biomedicines.

[144] Maki N., Suetsugu-Maki R., Tarui H., Agata K., del Rio-Tsonis K., Tsonis P.A. (2009). Expression of stem cell pluripotency factors during regeneration in newts. Dev. Dyn..

[145] Islam M.R., Nakamura K., Casco-Robles M.M., Kunahong A., Inami W., Toyama F., Maruo F., Chiba C. (2014). The newt reprograms mature RPE cells into a unique multipotent state for retinal regeneration. Sci. Rep..

[146] Kaneko J., Chiba C. (2009). Immunohistochemical analysis of Musashi-1 expression during retinal regeneration of adult newt. Neurosci. Lett..

[147] Makar’ev E.O., Zinov’eva R.D., Mitashov V.I. Expression of regulatory homeobox genes during retina regeneration in adult newts. Izv Akad Nauk Ser Biol..

[148] Markitantova Y.V., Avdonin P.P., Grigoryan E.N., Zinovieva R.D. (2010). Identification of the *pitx1* embryogenesis regulatory gene in a regenerating newt retina. Dokl. Biol. Sci..

[149] Casco-Robles M.M., Islam M.R., Inami W., Tanaka H.V., Kunahong A., Yasumuro H., Hanzawa S., Casco-Robles R.M., Toyama F., Maruo F. (2016). Turning the fate of reprogramming cells from retinal disorder to regeneration by Pax6 in newts. Sci. Rep..

[150] Kaneko Y., Hirota K., Matsumoto G., Hanyu Y. (2001). Expression pattern of a newt Notch homologue in regenerating newt retina. Brain Res. Dev. Brain Res..

[151] Nakamura K., Chiba C. (2007). Evidence for Notch signaling involvement in retinal regeneration of adult newt. Brain Res..

[152] Mercer S.E., Cheng C.H., Atkinson D.L., Krcmery J., Guzman C.E., Kent D.T., Zukor K., Marx K.A., Odelberg S.J., Simon H.G. (2012). Multi-tissue microarray analysis identifies a molecular signature of regeneration. PLoS ONE.

[153] Sherpa T., Lankford T., McGinn T.E., Hunter S.S., Frey R.A., Sun C., Ryan M., Robison B.D., Stenkamp D.L. (2014). Retinal regeneration is facilitated by the presence of surviving neurons. Dev. Neurobiol..

[154] Spence J.R., Aycinena J.-C., del Rio-Tsonis K. (2007). FGF-Hedgehog Interdependence During Retina Regeneration. Dev. Dyn..

[155] Qin Z., Kidd A.R., Thomas J.L., Poss K.D., Hyde D.R., Raymond P.A., Thummel R. (2011). FGF signaling regulates rod photoreceptor cell maintenance and regeneration zebrafish. Exp. Eye Res..

[156] Fukui L., Henry J.J. (2011). FGF signaling is required for lens regeneration in *Xenopus laevis*. Biol. Bull..

[157] Susaki K., Chiba C. (2007). MEK mediates in vitro neural transdifferentiation of the adult newt retinal pigment epithelium cells: Is FGF2 an induction factor?. Cell Res..

[158] Markitantova Y.V., Avdonin P.P., Grigoryan E.N. (2014). FGF2 signaling pathway components in tissues of the posterior eye sector in the adult newt *Pleurodeles waltl*. Biol. Bull..

[159] Dvoriantchikova D., Seemungal R.J., Ivanov D. (2019). The epigenetic basis for the impaired ability of adult murine retinal pigment epithelium cells to regenerate retinal tissue. Sci. Rep..

[160] Yoshii C., Ueda Y., Okamoto M., Araki M. (2007). Neural retinal regeneration in the anuran amphibian *Xenopus laevis* post-metamorphosis: Transdifferentiation of retinal pigmented epithelium regenerates the neural retina. Dev. Biol..

[161] Vergara M.N., del Rio-Tsonis K. (2009). Retina regeneration in the *Xenopus laevis* tadpole: A new model system. Mol. Vis..

[162] Arresta E., Bernardini S., Bernardini E., Filoni S., Cannata S.M. (2005). Pigmented epithelium to retinal transdifferentiation and Pax6 expression in larval *Xenopus laevis*. J. Exp. Zool. A Comp. Exp. Biol..

[163] Nabeshima A., Nishibayashi C., Ueda Y., Ogino H., Araki M. (2013). Loss of cell-extracellular matrix interaction triggers retinal regeneration accompanied by Rax and Pax6 activation. Genesis.

[164] Andreazzoli M., Gestri G., Angeloni D., Menna E., Barsacchi G. (1999). Role of Xrx1 in Xenopus eye and anterior brain development. Development..

[165] Sakaguchi D.S., Janick L.M., Reh T.A. (1997). Basic Fibroblast Growth Factor (FGF-2) induced transdifferentiation of retinal pigment epithelium: Generation of retinal neurons and glia. Dev. Dyn..

[166] Naitoh H., Suganuma Y., Ueda Y., Sato T., Hiramuki Y., Fujisawa-Sehara A., Taketani S., Araki M. (2017). Upregulation of matrix metalloproteinase triggers transdifferentiation of retinal pigmented epithelial cells in *Xenopus laevis*: A Link between inflammatory response and regeneration. Dev. Neurobiol..

[167] Suzuki N., Ochi H. (2020). Regeneration enhancers: A clue to reactivation of developmental genes. Dev. Growth Differ..

[168] Coulombre J.L., Coulombre A.J. (1965). Regeneration of neural retina from the pigmented epithelium in the chick embryo. Dev. Biol..

[169] Park C.M., Hollenberg M.J. (1993). Growth factor-induced retinal regeneration in vivo. Int. Rev. Cytol..

[170] Haynes T., Luz-Madrigal A., Reis E.S., Echeverri Ruiz N.P., Grajales-Esquivel E., Tzekou A., Tsonis P.A., Lambris J.D., del Rio-Tsonis K. (2013). Complement anaphylatoxin C3a is a potent inducer of embryonic chick retina regeneration. Nat. Commun..

[171] Zhu J., Luz-Madrigal A., Haynes T., Zavada J., Burke A.K., del Rio-Tsonis K. (2014). Catenin Inactivation is a Pre-Requisite for Chick Retina Regeneration. PLoS ONE.

[172] Spence J.R., Madhavan M., Aycinena J.-C., del Rio-Tsonis K. (2007). Retina regeneration in the chick embryo is not induced by spontaneous Mitf downregulation but requiRes. FGF/FGFR/MEK/Erk dependent upregulation of Pax6. Mol. Vis..

[173] Steinfeld J., Steinfeld I., Bausch A., Coronato N., Hampel M.-L., Depner H., Layer P.G., Vogel-Höpker A. (2017). BMP-induced reprogramming of the neural retina into retinal pigment epithelium requiRes. Wnt signalling. Biol. Open.

[174] Tangeman J.A., Luz-Madrigal A., Sreeskandarajan S., Grajales-Esquivel E., Liu L., Liang C.H., Tsonis P.A., del Rio-Tsonis K. (2021). Transcriptome Profiling of Embryonic Retinal Pigment Epithelium Reprogramming. Genes.

[175] Luz-Madrigal A., Grajales-Esquivel E., Tangeman J., Kosse S., Liu L., Wang K., Fausey A., Liang C., Tsonis P.A., del Rio-Tsonis K. (2020). DNA demethylation is a driver for chick retina regeneration. Epigenetics.

[176] Xia H., Krebs M.P., Kaushal S., Scott E.W. (2011). Enhanced retinal pigment epithelium regeneration after injury in MRL/MpJ mice. Exp. Eye Res..

[177] Kampik D., Basche M., Luhmann U.F.O., Nishiguchi K.M., Williams J.A.E., Greenwood J., Moss S.E., Han H., Azam S., Duran Y. (2017). In situ regeneration of retinal pigment epithelium by gene transfer of E2F2: A potential strategy for treatment of macular degenerations. Gene Ther..

[178] Grigoryan E.N., Novikova Y.P., Kilina O.V., Philippov P.P. (2007). New method of in vitro culturing of pigment retinal epithelium in the structure of the posterior eye sector of adult rat. Bull. Exp. Biol. Med..

[179] Hadziahmetovic M., Dentchev T., Song Y., Haddad N., He X., Hahn P., Pratico D., Wen R., Harris Z.L., Lambris J.D. (2008). Ceruloplasmin/ hephaestin knockout mice model morphologic and molecular features. of AMD. Investig. Ophthalmol. Vis. Sci..

[180] Longbottom R., Fruttiger M., Douglas R.H., Martinez-Barbera J.P., Greenwood J., Moss S.E. (2009). Genetic ablation of retinal pigment epithelial cells reveals the adaptive response of the epithelium and impact on photoreceptors. Proc. Natl. Acad. Sci. USA.

[181] Kolomeyer A.M., Zarbin M.A. (2014). Trophic factors in the pathogenesis and therapy for retinal degenerative diseases. Surv. Ophthalmol..

[182] Grigoryan E.N., Davies J. (2012). Shared triggering mechanisms of retinal regeneration in lower vertebrates and retinal rescue in higher ones. Tissue Regeneration—From Basic Biology to Clinical Application.

[183] Al-Hussaini H., Kam J.H., Vugler A., Semo M., Jeffery G. (2008). Mature retinal pigment epithelium cells are retained in the cell cycle and proliferate in vivo. Mol. Vis..

[184] Kokkinopoulos I., Shahabi G., Colman A., Jeffery G. (2011). Mature peripheral RPE cells have an intrinsic capacity to proliferate; a potential regulatory mechanism for age-related cell loss. PLoS ONE.

[185] Kirchhof B., Sorgente N. (1989). Pathogenesis of proliferative vitreoretinopathy. Modulation of retinal pigment epithelial cell functions by vitreous and macrophages. Dev. Ophthalmol..

[186] Abe T., Sato M., Tamai M. (1998). Dedifferentiation of the retinal pigment epithelium compared to the proliferative membranes of proliferative vitreoretinopathy. Curr. Eye Res..

[187] Tamiya S., Liu L., Kaplan H.J. (2010). Epithelial-mesenchymal transition and proliferation of retinal pigment epithelial cells initiated upon loss of cell-cell contact. Investig. Ophthalmol. Vis. Sci..

[188] Wang L.C., Hung K.H., Hsu C.C., Chen S.J., Li W.Y., Lin T.C. (2015). Assessment of retinal pigment epithelial cells in epiretinal membrane formation. J. Chin. Med. Assoc..

[189] Wu J., Chen X., Liu X., Huang S., He C., Chen B., Liu Y. (2018). Autophagy regulates TGF-beta2-induced epithelial-mesenchymal transition in human retinal pigment epithelium cells. Mol. Med. Rep..

[190] Garweg J.G., Tappeiner C., Halberstadt M. (2013). Pathophysiology of proliferative vitreoretinopathy in retinal detachment. Surv. Ophthalmol..

[191] Tamiya S., Kaplan H.J. (2016). Role of epithelial-mesenchymal transition in proliferative vitreoretinopathy. Exp. Eye Res..

[192] Han J.W., Lyu J., Park Y.J., Jang S.-Y., Park T.K. (2015). Wnt/β-catenin signaling mediates regeneration of retinal pigment epithelium after laser photocoagulation in mouse eye. Investig. Ophthalmol. Vis. Sci..

[193] Kent D., Sheridan C. (2003). Choroidal neovascularization: A wound healing perspective. Mol. Vis..

[194] Ishikawa K., Kannan R., Hinton D.R. (2016). Molecular mechanisms of subretinal fibrosis in age-related macular degeneration. Exp. Eye Res..

[195] Philp N.J., Nachmias V.T. (1987). Polarized distribution of integrin and fibronectin in retinal pigment epithelium. Investig. Ophthalmol. Vis. Sci..

[196] Huang X., Wei Y., Ma H., Zhang S. (2012). Vitreous-induced cytoskeletal rearrangements via the Rac1 GTPase-dependent signaling pathway in human retinal pigment epithelial cells. Biochem. Biophys. Res. Commun..

[197] Thiery J.P., Sleeman J.P. (2006). Complex networks orchestrate epithelial-mesenchymal transitions. Nat. Rev. Mol. Cell Biol..

[198] Imamichi Y., Menke A. (2007). Signaling pathways involved in collagen-induced disruption of the E-cadherin complex during epithelial-mesenchymal transition. Cells Tissues Organs.

[199] Sheridan C., Hiscott P., Grierson I., Kirchhof B., Wong D. (2005). Retinal Pigment Epithelium Differentiation and Dedifferentiation. Vitreo-Retinal Surgery.

[200] Vadigepalli R., Chakravarthula P., Zak D.E., Schwaber J.S., Gonye G.E. (2003). PAINT: A promoter analysis and interaction network generation tool for gene regulatory network identification. OMICS.

[201] Nazarieh M., Wiese A., Will T., Hamed M., Helms V. (2016). Identification of key player genes in gene regulatory networks. BMC Syst. Biol..

[202] Benayoun B.A., Caburet S., Veitia R.A. (2011). Forkhead transcription factors: Key players in health and disease. Trends Genet..

[203] Chen X., Muller G.A., Quaas M., Fischer M., Han N., Stutchbury B., Sharrocks A.D., Engeland K. (2013). The forkhead transcription factor FOXM1 controls cell cycle-dependent gene expression through an atypical chromatin binding mechanism. Mol. Cell Biol..

[204] Choudhary P., Dodsworth B.T., Sidders B., Gutteridge A., Michaelides C., Duckworth J.K.L., Whiting P.J., Benn C.L. (2015). A FOXM1 dependent mesenchymal-epithelial transition in retinal pigment epithelium cells. PLoS ONE.

[205] Shu D.Y., Butcher E., Saint-Geniez M. (2020). EMT and EndMT: Emerging Roles in Age-Related Macular Degeneration. Int. J. Mol. Sci..

[206] Hua X., Liu X., Ansari D.O., Lodish H.F. (1998). Synergistic cooperation of TFE3 and smad proteins in TGF-beta induced transcription of the plasminogen activator inhibitor-1 gene. Genes Dev..

[207] Kang Y., Massague J. (2004). Epithelial-mesenchymal transitions: Twist in development and metastasis. Cell.

[208] Pratt C.H., Vadigepalli R., Chakravarthula P., Gonye G.E., Philp N.J., Grunwald G.B. (2008). Transcriptional regulatory network analysis during epithelial-mesenchymal transformation of retinal pigment epithelium. Mol. Vis..

[209] Kaneko H., Terasaki H. (2017). Biological Involvement of MicroRNAs in Proliferative Vitreoretinopathy. Transl. Vis. Sci. Technol..

[210] Toro M.D., Reibaldi M., Avitabile T., Bucolo C., Salomone S., Rejdak R., Nowomiejska K., Tripodi S., Posarelli C., Ragusa M. (2020). MicroRNAs in the Vitreous Humor of Patients with Retinal Detachment and a Different Grading of Proliferative Vitreoretinopathy: A Pilot Study. Transl. Vis. Sci. Technol..

[211] Boles N.C., Fernandes M., Swigut T., Srinivasan R., Schiff L., Rada-Iglesias A., Wang Q., Saini J.S., Kiehl T., Stern J.H. (2020). Epigenomic and Transcriptomic Changes During Human RPE EMT in a Stem Cell Model of Epiretinal Membrane Pathogenesis and Prevention by Nicotinamide. Stem Cell Rep..

[212] Saika S., Yamanaka O., Okada Y., Tanaka S., Miyamoto T., Sumioka T., Kitano A., Shirai K., Ikeda K. (2009). TGF in fibroproliferative diseases in the eye. Front. Biosci..

[213] Xu J., Lamouille S., Derynck R. (2009). TGF-beta-induced epithelial to mesenchymal transition. Cell Res..

[214] Chen Z., Shao Y., Li X. (2015). The roles of signaling pathways in epithelial-to-mesenchymal transition of PVR. Mol. Vis..

[215] Dai Y., Dai C., Sun T. (2020). Inflammatory mediators of proliferative vitreoretinopathy: Hypothesis and review. Int. Ophthalmol..

[216] Chaudhary R., Scott R.A.H., Wallace G., Berry M., Logan A., Blanch R.J. (2020). Inflammatory and Fibrogenic Factors in Proliferative Vitreoretinopathy Development. Transl. Vis. Sci. Technol..

[217] Kita T., Hata Y., Arita R., Kawahara S., Miura M., Nakao S., Mochizuki Y., Enaida H., Goto Y., Shimokawa H. (2008). Role of TGF- β in proliferative vitreoretinal diseases and ROCK as a therapeutic target. Proc. Natl. Acad. Sci. USA.

[218] Korthagen N.M., van Bilsen K., Swagemakers S.M., van de Peppel J., Bastiaans J., van der Spek P.J., van Hagen P.M., Dik W.A. (2015). Retinal pigment epithelial cells display specific transcriptional responses upon TNF-alpha stimulation. Br. J. Ophthalmol..

[219] Yan X., Liu Z., Chen Y. (2009). Regulation of TGF-signaling by Smad7. Acta Biochim. Biophys. Sin..

[220] Schiff L., Boles N.C., Fernandes M., Nachmani B., Gentile R., Blenkinsop T.A. (2019). P38 inhibition reverses TGFb1 and TNFa induced contraction in a model of proliferative vitreoretinopathy. Commun. Biol..

[221] Ishida W., Mori Y., Lakos G., Sun L., Shan F., Bowes S., Josiah S., Lee W.C., Singh J., Ling L.E. (2006). Intracellular TGF-β receptor blockade abrogates smad-dependent fibroblast activation in vitro and in vivo. J. Investig. Dermatol..

[222] Pannu J., Nakerakanti S., Smith E., Ten Dijke P., Trojanowska M. (2007). Transforming growth factor-beta receptor type I-dependent fibrogenic gene program is mediated via activation of Smad1 and ERK1/2 pathways. J. Biol. Chem..

[223] Amemiya K., Haruta M., Takahashi M., Kosaka M., Eguchi G. (2004). Adult human retinal pigment epithelial cells capable of differentiating into neurons. Biochem. Biophys. Res. Commun..

[224] Engelhardt M., Bogdahn U., Aigner L. (2005). Adult retinal pigment epithelium cells express neural progenitor properties and the neuronal precursor protein doublecortin. Brain Res..

[225] Milyushina L.A., Verdiev B.I., Kuznetsova A.V., Aleksandrova M.A. (2012). Expression of multipotent and retinal markers in pigment epithelium of adult human in vitro. Bull. Exp. Biol. Med..

[226] Li S., Zhang H., Wang A., Liu Y., Liu H., Yue F., Abulaiti X., Zhang C., Li L. (2019). Differentiation of adult human retinal pigment epithelial cells into dopaminergic-like cells in vitro and in the recipient monkey brain. Mol. Med..

[227] Sakami S., Etter P., Reh T.A. (2008). Activin signaling limits the competence for retinal regeneration from the pigmented epithelium. Mech. Dev..

[228] Milyushina L.A., Kuznetsova A.V., Grigoryan E.N., Aleksandrova M.A. (2011). Phenotypic plasticity of retinal pigment epithelium cells of adult human eye in vitro. Bull. Exp. Biol. Med..

[229] Kuznetsova A.V., Kurinov A.M., Aleksandrova M.A. (2014). Cell models to study regulation of cell transformation in pathologies of retinal pigment epithelium. J. Ophthalmol..

[230] Shafei E.V., Kurinov A.M., Kuznetsova A.V., Aleksandrova M.A. (2017). Reprogramming of human retinal pigment epithelial cells under the effect of bFGF in vitro. Bull. Exp. Biol. Med..

[231] Burke J.M. (2008). Epithelial phenotype and the RPE: Is the answer blowing in the Wnt?. Prog. Retin. Eye Res..

[232] Chen F., Liu X., Chen Y., Liu J.Y., Lu H., Wang W., Lu X., Dean K.C., Gao L., Kaplan H.J. (2020). Sphere-induced reprogramming of RPE cells into dual-potential RPE stem-like cells. EBioMedicine.

[233] Salero E., Blenkinsop T.A., Corneo B., Harris A., Rabin D., Stern J.H., Temple S. (2012). Adult human RPE can be activated into a multipotent stem cell that produces mesenchymal derivatives. Cell Stem Cell.

[234] Chen H.C., Zhu Y.T., Chen S.Y., Tseng S.C. (2012). Wnt signaling induces epithelial-mesenchymal transition with proliferation in ARPE-19 cells upon loss of contact inhibition. Lab. Investig..

[235] Saini J.S., Temple S., Stern J.H. (2016). Human Retinal Pigment Epithelium Stem Cell (RPESC). Adv. Exp. Med. Biol..

[236] Coffey P. (2012). Untapping the potential of human retinal pigmented epithelial cells. Cell Stem Cell.

[237] Pandey R.S., Krebs M.P., Bolisetty M.T., Charette J.R., Naggert J.K., Robson P., Nishina P.M., Carter G.W. (2022). Single-Cell RNA Sequencing Reveals Molecular Features of Heterogeneity in the Murine Retinal Pigment Epithelium. Int. J. Mol. Sci..

[238] Hu J., Bok D. (2001). A cell culture medium that supports the differentiation of human retinal pigment epithelium into functionally polarized monolayers. Mol. Vis..

[239] Blenkinsop T.A., Salero E., Stern J.H., Temple S. (2013). The culture and maintenance of functional retinal pigment epithelial monolayers from adult human eye. Methods Mol. Biol..

[240] Blenkinsop T.A., Saini J.S., Maminishkis A., Bharti K., Wan Q., Banzon T., Lotfi M., Davis J., Singh D., Rizzolo L.J. (2015). Human Adult Retinal Pigment Epithelial Stem Cell-Derived RPE Monolayers Exhibit Key Physiological Characteristics of Native Tissue. Investig. Ophthalmol. Vis. Sci..

[241] Samuel W., Jaworski C., Postnikova O.A., Kutty R.K., Duncan T., Tan L.X., Poliakov E., Lakkaraju A., Redmond T.M. (2017). Appropriately differentiated ARPE-19 cells regain phenotype and gene expression profiles similar to those of native RPE cells. Mol. Vis..

[242] Lenkowski J.R., Raymond P.A. (2014). Muller glia: Stem cells for generation and regeneration of retinal neurons in teleost fish. Prog. Retin. Eye Res..

[243] Goldman D. (2014). Muller glial cell reprogramming and retina regeneration. Nat. Rev. Neurosci..

[244] Hamon A., Roger J.E., Yang X.J., Perron M. (2016). Muller glial cell-dependent regeneration of the neural retina: An overview across vertebrate model systems. Dev. Dyn..

[245] Chohan A., Singh U., Kumar A., Kaur J. (2017). Müller stem cell dependent retinal regeneration. Clin. Chim. Acta.

[246] Gao H., Huang X., Chen X., Xu H. (2021). Müller Glia-Mediated Retinal Regeneration. Mol. Neurobiol..

[247] Martins R.R., Zamzam M., Tracey-White D., Moosajee M., Thummel R., Henriques C.M., MacDonald R.B. (2022). Müller Glia maintain their regenerative potential despite degeneration in the aged zebrafish retina. Aging Cell.

[248] Otteson D.C. (2017). Talkin’ about my (re)generation: The who of intrinsic retinal stem cells. Neuroscience.

[249] Devoldere J., Peynshaert K., De Smedt S.C., Remaut K. (2019). Müller cells as a target for retinal therapy. Drug Discov. Today.

[250] de Hoz R., Rojas B., Ramirez A.I., Salazar J.J., Gallego B.I., Triviño A., Ramírez J.M. (2016). Retinal macroglial responses in health and disease. BioMed Res. Int..

[251] Reichenbach A., Bringmann A. (2013). New functions of Müller cells. Glia.

[252] Cameron D.A. (2000). Cellular proliferation and neurogenesis in the injured retina of adult zebrafish. Vis. Neurosci..

[253] Thummel R., Kassen S.C., Enright J.M., Nelson C.M., Montgomery J.E., Hyde D.R. (2008). Characterization of Müller glia and neuronal progenitors during adult zebrafish retinal regeneration. Exp. Eye Res..

[254] Nagashima M., Barthel L.K., Raymond P.A. (2013). A self-renewing division of zebrafish Müller glial cells generates neuronal progenitors that require N-cadherin to regenerate retinal neurons. Development.

[255] Nagashima M., Hitchcock P. (2021). Inflammation Regulates the Multi-Step Process of Retinal Regeneration in Zebrafish. Cells.

[256] Lahne M., Nagashima M., Hyde D.R., Hitchcock P.F. (2020). Reprogramming Müller Glia to Regenerate Retinal Neurons. Annu. Rev. Vis. Sci..

[257] Fischer A.J., McGuire C.R., Dierks B.D., Reh T.A. (2002). Insulin and fibroblast growth factor 2 activate a neurogenic program in Müller glia of the chicken retina. J. Neurosci..

[258] Todd L., Suarez L., Quinn C., Fischer A.J. (2018). Retinoic Acid-signaling regulates the proliferative and neurogenic capacity of Müller glia-derived progenitor cells in the avian retina. Stem Cells.

[259] Blackshaw S., Harpavat S., Trimarchi J., Cai L., Huang H., Kuo W.P., Weber G., Lee K., Fraioli R.E., Cho S.H. (2004). Genomic analysis of mouse retinal development. PLoS Biol..

[260] Roesch K., Jadhav A.P., Trimarchi J.M., Stadler M.B., Roska B., Sun B.B., Cepko C.L. (2008). The transcriptome of retinal Müller glial cells. J. Comp. Neurol..

[261] Too L.K., Gracie G., Hasic E., Iwakura J.H., Cherepanoff S. (2017). Adult human retinal Müller glia display distinct peripheral and macular expression of CD117 and CD44 stem cell-associated proteins. Acta Histochem..

[262] Bhatia B., Jayaram H., Singhal S., Jones M.F., Limb G.A. (2011). Differences between the neurogenic and proliferative abilities of Müller glia with stem cell characteristics and the ciliary epithelium from the adult human eye. Exp. Eye Res..

[263] Bringmann A., Wiedemann P. (2012). Muller glial cells in retinal disease. Ophthalmologica.

[264] Bringmann A., Iandiev I., Pannicke T., Wurm A., Hollborn M., Wiedemann P., Osborne N.N., Reichenbach A. (2009). Cellular signaling and factors involved in Muller cell gliosis: Neuroprotective and detrimental effects. Prog. Retin. Eye Res..

[265] Sardar Pasha S.P.B., Münch R., Schäfer P., Oertel P., Sykes A.M., Zhu Y., Karl M.O. (2017). Retinal cell death dependent reactive proliferative gliosis in the mouse retina. Sci. Rep..

[266] Bringmann A., Francke M., Reichenbach A. (2003). Müller Cells in Retinopathies. Adv. Mol. Cell Biol..

[267] Bringmann A., Pannicke T., Grosche J., Francke M., Wiedemann P., Skatchkov S.N., Osborne N.N., Reichenbach A. (2006). Müller cells in the healthy and diseased retina. Prog. Retin. Eye Res..

[268] Close J.L., Liu J., Gumuscu B., Reh T.A. (2006). Epidermal growth factor receptor expression regulates proliferation in the postnatal rat retina. Glia.

[269] Osakada F., Ooto S., Akagi T., Mandai M., Akaike A., Takahashi M. (2007). Wnt signaling promotes regeneration in the retina of adult mammals. J. Neurosci..

[270] Wan J., Zheng H., Xiao H.L., She Z.J., Zhou G.M. (2007). Sonic hedgehog promotes stem-cell potential of Müller glia in the mammalian retina. Biochem. Biophys. Res. Commun..

[271] Del Debbio C.B., Balasubramanian S., Parameswaran S., Chaudhuri A., Qiu F., Ahmad I. (2010). Notch and Wnt signaling mediated rod photoreceptor regeneration by Müller cells in adult mammalian retina. PLoS ONE.

[272] Liu B., Hunte D.J., Rooker S., Chan A., Paulus Y.M., Leucht P., Nomoto H.Y., Helms J.A. (2013). Wnt signaling promotes Müller cell proliferation and survival after injury. Investig. Ophthalmol. Vis. Sci..

[273] Limb G.A., Salt T.E., Munro P.M., Moss S.E., Khaw P.T. (2002). In vitro characterization of a spontaneously immortalized human Muller cell line (MIO-M1). Investig. Ophthalmol. Vis. Sci..

[274] Lawrence J.M., Singhal S., Bhatia B., Keegan D.J., Reh T.A., Luthert P.J., Khaw P.T., Limb G.A. (2007). MIO-M1 cells and similar muller glial cell lines derived from adult human retina exhibit neural stem cell characteristics. Stem Cells.

[275] Coughlin B.A., Feenstra D.J., Mohr S. (2017). Müller cells and diabetic retinopathy. Vis. Res..

[276] Walker R.J., Anderson N.M., Jiang Y., Bahouth S., Steinle J.J. (2011). Role of β-Adrenergic Receptor Regulation of TNF-α and Insulin Signaling in Retinal Müller Cells. Investig. Ophthalmol. Vis. Sci..

[277] Insua M.F., Simón M.V., Garelli A., de Los Santos B., Rotstein N.P., Politi L.E. (2008). Trophic factors and neuronal interactions regulate the cell cycle and Pax6 expression in Müller stem cells. J. Neurosci. Res..

[278] Wohl S.G., Reh T.A. (2016). The microRNA expression profile of mouse Müller glia in vivo and in vitro. Sci. Rep..

[279] Kara N., Kent M.R., Didiano D., Rajaram K., Patton J.G. (2019). The miR-216a-Dot1l Regulatory Axis Is Necessary and Sufficient for Muller Glia Reprogramming during Retina Regeneration. Cell Rep..

[280] Wohl S.G., Hooper M.J., Reh T.A. (2019). MicroRNAs miR-25, let-7 and miR-124 regulate the neurogenic potential of Müller glia in mice. Development.

[281] Suzuki F., Okuno M., Tanaka T., Sanuki R. (2020). Overexpression of neural miRNAs miR-9/9* and miR-124 suppresses differentiation to Müller glia and promotes differentiation to neurons in mouse retina in vivo. Genes Cells.

[282] Greene K.M., Stamer W.D., Liu Y. (2022). The role of microRNAs in glaucoma. Exp. Eye Res..

[283] Ueno K., Iwagawa T., Ochiai G., Koso H., Nakauchi H., Nagasaki M., Suzuki Y., Watanabe S. (2017). Analysis of Muller glia specific genes and their histone modification using Hes1-promoter driven EGFP expressing mouse. Sci. Rep..

[284] Dvoriantchikova G., Seemungal R.J., Ivanov D. (2019). Development and epigenetic plasticity of murine Müller glia. Biochim. Biophys. Acta Mol. Cell Res..

[285] Powell C., Grant A.R., Cornblath E., Goldman D. (2013). Analysis of DNA methylation reveals a partial reprogramming of the Muller glia genome during retina regeneration. Proc. Natl. Acad. Sci. USA.

[286] Jorstad N.L., Wilken M.S., Grimes W.N., Wohl S.G., Vanden Bosch L.S., Yoshimatsu T., Wong R.O., Rieke F., Reh T.A. (2017). Stimulation of functional neuronal regeneration from Müller glia in adult mice. Nature.

[287] Fausett B.V., Gumerson J.D., Goldman D. (2008). The proneural basic helix-loop-helix gene *ascl1a* is required for retina regeneration. J. Neurosci..

[288] Hoang T., Wang J., Boyd P., Wang F., Santiago C., Jiang L., Yoo S., Lahne M., Todd L.J., Jia M. (2020). Gene regulatory networks controlling vertebrate retinal regeneration. Science.

[289] Bonilla-Pons S.A., Nakagawa S., Bahima E.G., Fernandez-Blanco A., Pesaresi M., D’Antin J.C., Sebastian-Perez R., Greco D., Domínguez-Sala E., Gómez-Riera R. (2022). Müller glia fused with adult stem cells undergo neural differentiation in human retinal models. eBioMedicine.

[290] Sanges D., Simonte G., Di Vicino U., Romo N., Pinilla I., Nicolás M., Cosma M.P. (2016). Reprogramming Muller glia via in vivo cell fusion regenerates murine photoreceptors. J. Clin. Investig..

[291] Clevers H., Loh K.M., Nusse R. (2014). Stem cell signaling. An integral program for tissue renewal and regeneration: Wnt signaling and stem cell control. Science.

[292] Liu Z., Mikrani R., Zubair H.M., Taleb A., Naveed M., Baig M.F.A., Zhang Q., Li C., Habib M., Cui X. (2020). Systemic and local delivery of mesenchymal stem cells for heart renovation: Challenges and innovations. Eur. J. Pharmacol..

[293] González Fleitas M.F., Devouassoux J.D., Aranda M.L., Calanni J.S., Chianelli M.S., Dorfman D., Rosenstein R.E. (2020). Enriched environment provides neuroprotection against experimental glaucoma. J. Neurochem..

